# Effects of Diet on Resource Utilization by a Model Human Gut Microbiota Containing *Bacteroides cellulosilyticus* WH2, a Symbiont with an Extensive Glycobiome

**DOI:** 10.1371/journal.pbio.1001637

**Published:** 2013-08-20

**Authors:** Nathan P. McNulty, Meng Wu, Alison R. Erickson, Chongle Pan, Brian K. Erickson, Eric C. Martens, Nicholas A. Pudlo, Brian D. Muegge, Bernard Henrissat, Robert L. Hettich, Jeffrey I. Gordon

**Affiliations:** 1 Center for Genome Sciences and Systems Biology, Washington University School of Medicine, St. Louis, Missouri, United States of America; 2 Graduate School of Genome Science and Technology, University of Tennessee–Oak Ridge National Laboratory, Knoxville, Tennessee, United States of America; 3 Chemical Sciences Division, Oak Ridge National Laboratory, Oak Ridge, Tennessee, United States of America; 4 Department of Microbiology and Immunology, University of Michigan Medical School, Ann Arbor, Michigan, United States of America; 5 Architecture et Fonction des Macromolécules Biologiques, CNRS and Aix-Marseille University, Marseille, France; University of California Davis, United States of America

## Abstract

The human gut microbiota is an important metabolic organ, yet little is known about how its individual species interact, establish dominant positions, and respond to changes in environmental factors such as diet. In this study, gnotobiotic mice were colonized with an artificial microbiota comprising 12 sequenced human gut bacterial species and fed oscillating diets of disparate composition. Rapid, reproducible, and reversible changes in the structure of this assemblage were observed. Time-series microbial RNA-Seq analyses revealed staggered functional responses to diet shifts throughout the assemblage that were heavily focused on carbohydrate and amino acid metabolism. High-resolution shotgun metaproteomics confirmed many of these responses at a protein level. One member, *Bacteroides cellulosilyticus* WH2, proved exceptionally fit regardless of diet. Its genome encoded more carbohydrate active enzymes than any previously sequenced member of the Bacteroidetes. Transcriptional profiling indicated that *B. cellulosilyticus* WH2 is an adaptive forager that tailors its versatile carbohydrate utilization strategy to available dietary polysaccharides, with a strong emphasis on plant-derived xylans abundant in dietary staples like cereal grains. Two highly expressed, diet-specific polysaccharide utilization loci (PULs) in *B. cellulosilyticus* WH2 were identified, one with characteristics of xylan utilization systems. Introduction of a *B. cellulosilyticus* WH2 library comprising >90,000 isogenic transposon mutants into gnotobiotic mice, along with the other artificial community members, confirmed that these loci represent critical diet-specific fitness determinants. Carbohydrates that trigger dramatic increases in expression of these two loci and many of the organism's 111 other predicted PULs were identified by RNA-Seq during *in vitro* growth on 31 distinct carbohydrate substrates, allowing us to better interpret *in vivo* RNA-Seq and proteomics data. These results offer insight into how gut microbes adapt to dietary perturbations at both a community level and from the perspective of a well-adapted symbiont with exceptional saccharolytic capabilities, and illustrate the value of artificial communities.

## Abstract 2

Artificial human gut microbial communities implanted into germ-free mice provide insights into how species-level responses to changes in diet give rise to community-level structural and functional reconfiguration and how types of bacteria prioritize use of available nutrients *in vivo*.

## Author Summary

Our intestines are populated by an almost unimaginably large number of microbial cells, most of which are bacteria. This species assemblage operates as a microbial metabolic organ, performing myriad tasks that contribute to our well-being, including processing components of our diet. The way this incredible machine assembles itself and operates remains mysterious. One approach to understanding its properties is to create artificial communities composed of a limited number of sequenced human gut bacterial species and to install them in the guts of germ-free mice that are then fed different diets. In this report, we adopt this approach. We describe the genome sequence of a new gut bacterial isolate, *Bacteroides cellulosilyticus* WH2, which is equipped with an unprecedented number of carbohydrate active enzymes. Deploying four different “omics” technologies, we characterize the response to diet, the relative stability, and the temporal dynamics of a 12-species artificial bacterial assemblage (including *B. cellulosilyticus* WH2) implanted in germ-free mouse guts. We also combine high-throughput substrate utilization screens and RNA-Seq to generate reference data analogous to a “Rosetta stone” in order to decipher what types of carbohydrates *B. cellulosilyticus* encounters and uses within the gut, and how it interacts with other organisms that have similar and/or distinct “professions.” This work sets the stage for future ecological and metabolic studies of more complex assemblages that more fully emulate the properties of our native gut communities.

## Introduction

A growing body of evidence indicates that the tens of trillions of microbial cells that inhabit our gastrointestinal tracts extend our biological capabilities in important ways. Microbial enzymes process many compounds that would otherwise pass through our intestines unaltered [1], and cases of particular nutrient substrates favoring the growth of particular taxa are being reported [2]–[5]. Changes in diet are therefore expected to lead to changes in the composition and function of the microbiota [6]–[10]. However, our understanding of diet–microbiota interactions at a mechanistic level is still in its infancy.

The absence of a complete catalog of the microbial strains and associated genome sequences that comprise a given microbiota complicates efforts to describe how particular dietary substrates influence individual taxa, how taxa cooperate/compete to utilize nutrients, and how these many interactions in aggregate lead to emergent host phenotypes. Gnotobiotic mice colonized with defined consortia of sequenced human gut microbes, on the other hand, provide an *in vivo* model of the microbiota in which the identity of all taxa and genes comprising the system are known. Within these assemblages, expressed mRNAs and proteins can be attributed to their genome, gene, and species of origin, and findings of interest can be pursued in follow-up *in vitro* or *in vivo* experiments. These systems also afford an opportunity to tightly control experimental variables to a degree not possible in human studies and have proven useful in studying microbial invasion, microbe–microbe interactions, and the metabolic roles of key ecological guilds [11]–[15]. Studies aiming to better understand community-level assembly, resilience, and adaptation are therefore likely to benefit from a focus on such defined systems. However, the limited taxonomic and functional representation within artificial communities of modest complexity requires that caution be exercised when extrapolating results to more complex, naturally occurring gut communities (see *Prospectus*).

Culture-independent surveys of the healthy adult gut microbiota consistently conclude that it is composed primarily of members of two bacterial phyla, the Bacteroidetes and Firmicutes [16]–[21]. The dominance of these two bacterial phyla suggests that their representatives in the human gut are exquisitely adapted to its dynamic conditions, which include a constantly evolving nutrient environment. Members of the genus *Bacteroides* are known to be adept at utilizing both plant- and host-derived polysaccharides [22]. Comparisons of available *Bacteroides* genomes with those from other gut species indicate that the former are enriched in genes involved in the acquisition and metabolism of various glycans, including glycoside hydrolases (GHs) and polysaccharide lyases (PLs), as well as linked environmental sensors that control their expression (e.g., hybrid two-component systems, extracytoplasmic function (ECF) sigma factors and anti-sigma factors). Many of these genes are organized into polysaccharide utilization loci (PULs) that are distributed throughout the genome [23],[24]. Recent studies have focused on better understanding the evolution, specificity, and regulation of PULs in the genomes of species like *Bacteroides thetaiotaomicron* and *Bacteroides ovatus* [25],[26]. Little is known, however, about the metabolic strategies adopted by multiple competing species in more complex communities, how dietary changes lead to reconfigurations in community structure through changes in individual species, or whether dietary context influences which genes dominant species rely on to remain competitive with other microbes, including those genes that are components of PULs.

Here, we adopt a multifaceted approach to study an artificial community in gnotobiotic mice fed changing diets in order to better understand (i) the process by which such a community reconfigures itself structurally in response to changes in host diet; (ii) how aggregate community function, as judged by the metatranscriptome and metaproteome, is impacted when host diet is altered; (iii) how the metabolic strategies of its individual component microbes change, if at all, when the nutrient milieu is dramatically altered, with an emphasis on one prominent but understudied member of the human gut *Bacteroides*; and (iv) whether a microbe's metabolic versatility/flexibility correlates with competitive advantage in an assemblage containing related and unrelated species.

## Results and Discussion

### Sequencing the *Bacteroides cellulosilyticus* WH2 Genome

Though at least eight complete and 68 draft genomes of *Bacteroides* spp. are currently available [27], there are numerous examples of distinct clades within this genus where little genomic information exists. To further explore the genome space of one such clade, we obtained a human fecal isolate whose four 16S rRNA gene sequences indicate a close relationship to *Bacteroides cellulosilyticus* (Figure S1A,B). The genome of this isolate, which we have designated *B. cellulosilyticus* WH2, was sequenced deeply, yielding a high-quality draft assembly (23 contigs with an N50 value of 798,728 bp; total length of all contigs in the assembly, 7.1 Mb; Table S1). Annotation of its 5,244 predicted protein-coding genes using the carbohydrate active enzyme (CAZy) database [28] revealed an extraordinary complement of 503 CAZymes comprising 373 GHs, 23 PLs, 28 carbohydrate esterases (CEs), and 84 glycosyltransferases (GTs) (see Table S2 for all annotated genes in the *B. cellulosilyticus* WH2 genome predicted to have relevance to carbohydrate metabolism). One distinguishing feature of gut *Bacteroides* genomes is the substantial number of CAZymes they encode relative to those of other intestinal bacteria [29]. The *B. cellulosilyticus* WH2 CAZome is enriched in a number of GH families even when compared with prominent representatives of the gut Bacteroidetes (Figure S2A). When we expanded this comparison to include all 86 Bacteroidetes in the CAZy database, we found that the *B. cellulosilyticus* WH2 genome had the greatest number of genes for 19 different GH families, as well as genes from two GH families that had not previously been observed within a Bacteroidetes genome (Figure S2B). Altogether, *B. cellulosilyticus* WH2 has more GH genes at its disposal than any other Bacteroidetes species analyzed to date.

In *Bacteroides* spp., CAZymes are often located within PULs [30]. At a minimum, a typical PUL harbors a pair of genes with significant homology to the *susC* and *susD* genes of the starch utilization system (Sus) in *B. thetaiotaomicron* [30]–[32]. Other genes encoding enzymes capable of liberating oligo- and monosaccharides from a larger polysaccharide are also frequently present. The *susC*- and *susD*-like genes of these loci encode the proteins that comprise the main outer membrane binding and transport apparatus and thus represent key elements of these systems. A search of the *B. cellulosilyticus* WH2 genome for genes with strong homology to the *susC*- and *susD*-like genes in *B. thetaiotaomicron* VPI-5482 revealed an unprecedented number of *susC*/*D* pairs (a total of 118). Studies of other prominent *Bacteroides* spp. have found that the evolutionary expansion of these genes has played an important role in endowing the *Bacteroides* with the ability to degrade a wide range of host- and plant-derived polysaccharides [25],[33]. Analysis of deeply sampled adult human gut microbiota datasets indicates that *B. cellulosilyticus* strains are common, colonizing approximately 77% of 124 adult Europeans characterized in one study [18] and 62% of 139 individuals living in the United States examined in another survey [20]. We hypothesized that the apparent success of *B. cellulosilyticus* in the gut is derived in part from its substantial arsenal of genes involved in carbohydrate utilization.

### Measuring Changes in the Structural Configuration of a 12-Member Model Microbiota in Response to a Dietary Perturbation

**Figure 1:**
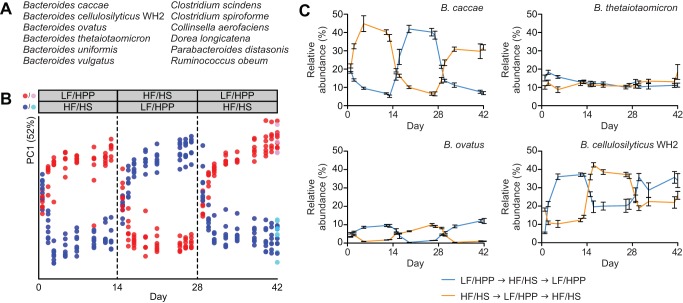
COPRO-Seq analysis of the structure of a 12-member artificial human gut microbial community as a function of diet and time. (A) The 12 bacterial species comprising the artificial community. (B) Principal coordinates analysis (PCoA) was applied to relative abundance data generated by COPRO-Seq from two experiments (E_1_, E_2_), each spanning 6 wk. Following colonization (day 0), mice were switched between two different diets at 2-wk intervals as described in Figure S3. COPRO-Seq data from E_1_ and E_2_ were ordinated in the same multidimensional space. For clarity, only data from E_2_ are shown here (for the E_1_ PCoA plot, see Figure S5A). Red/blue, feces; pink/cyan, cecal contents. (C) Proportional abundance data from E_1_ illustrating the impact of diet on fecal levels of a diet-sensitive strain with higher representation on HF/HS chow (*B. caccae*), a diet-sensitive strain with higher representation on LF/HPP chow (*B. ovatus*), a diet-insensitive strain with no obvious diet preference (*B. thetaiotaomicron*), and a diet-sensitive strain with a preference for the LF/HPP diet that also achieves a high level of representation on the HF/HS diet (*B. cellulosilyticus* WH2). Mean values ± SEM are shown. Plots illustrating changes in abundance over time for all species in both experiments are provided in Figure S4C.

To test the fitness of *B. cellulosilyticus* WH2 in relation to other prominent gut symbionts, and the importance of diet on its fitness, we carried out an experiment in gnotobiotic mice (experiment 1, “E_1_,” Figure S3). Two groups of 10–12-wk-old male germ-free C57BL/6J animals were moved to individual cages within gnotobiotic isolators (*n* = 7 animals/group). At day zero, each animal was colonized by oral gavage with an artificial community comprising 12 human gut bacterial species (Figure 1A, Table S3). Each species chosen for inclusion in this microbial assemblage met four criteria: (i) it was a member of one of three bacterial phyla routinely found in the human gut (i.e., Bacteroidetes, Firmicutes, or Actinobacteria), (ii) it was identified as a prominent member of the human gut microbiota in previous culture-independent surveys, (iii) it could be grown in the laboratory, and (iv) its genome had been sequenced to at least a high-quality draft level. Species were also selected for their functional attributes (as judged by their annotated gene content) in an effort to create an artificial community that was somewhat representative of a more complex human microbiota. For example, although more than half of the species in the assemblage were Bacteroidetes predicted to excel at the breakdown of polysaccharides, several were also prominent inhabitants of the human gut that are thought to have limited carbohydrate utilization capabilities (e.g., Firmicutes from Clostridium cluster XIVa). Some attributes for the 12 strains included in the artificial community are provided in Table S4.

For 2 wk, each treatment group was fed a standard low-fat/high-plant polysaccharide (LF/HPP) mouse chow, or a “Western”-like diet where calories are largely derived from fat, starch, and simple sugars (high-fat/high-sugar (HF/HS)) [12]. Over the course of 6 wk, diets were changed twice at 2-wk intervals, such that each group began and ended on the same diet, with an intervening 2-wk period during which the other diet was administered (Figure S3).

Using fecal DNA as a proxy for microbial biomass, the plant polysaccharide-rich LF/HPP diet supported 2- to 3-fold more total bacterial growth (primary productivity) despite its lower caloric density (3.7 kcal/g versus 4.5 kcal/g for the HF/HS diet; Figure S4A). The HF/HS diet contains carbohydrates that are easily metabolized and absorbed in the proximal intestine (sucrose, corn starch, and maltodextrin), with cellulose being the one exception (4% of the diet by weight versus 46.3% for the other carbohydrate sources). Thus, in mice fed the HF/HS diet, diet-derived simple sugars are likely to be rare in the distal gut where the vast majority of gut microbes reside; this may provide an advantage to those bacteria capable of utilizing other carbon sources (e.g., proteins/oligopeptides, host glycans). In mice fed the LF/HPP diet, on the other hand, plant polysaccharides that are indigestible by the host should provide a plentiful source of energy for saccharolytic members of the artificial community.

To evaluate the impact of each initial diet and subsequent diet switch on the structural configuration of the artificial community, we performed shotgun sequencing (community profiling by sequencing; COPRO-Seq) [11] of DNA isolated from fecal samples collected throughout the course of the experiment, as well as cecal contents collected at sacrifice. The relative abundances of the species in each sample (defined by the number of sequencing reads that could be unambiguously assigned to each microbial genome after adjusting for genome uniqueness) were subjected to ordination by principal coordinates analysis (PCoA) (Figure S5A). As expected, diet was found to be the predominant explanatory variable for observed variance (see separation along principal coordinate 1, “PC1,” which accounts for 52% of variance). The overall structure of the artificial community achieved quasi-equilibrium before the midpoint of the first diet phase, as evidenced by the lack of any significant movement along PC1 after day five. A structural reconfiguration also took place over the course of ∼5 d following transition to the second diet phase. Notably, the two treatment groups underwent a near-perfect inversion in their positions along PC1 after the first diet switch; the artificial community in animals switched from a LF/HPP to HF/HS diet took on a structure like that which arose by the end of the first diet phase in animals consuming the HF/HS diet, and vice versa. The second diet switch from phase 2 to 3 resulted in a similar movement along PC1 in the opposite direction, indicating a reversion of the artificial community's configuration to its originally assembled structure in each treatment group. These results, in addition to demonstrating the significant impact of these two diets on the structure of this 12-member artificial human gut community, also suggest that an assemblage of this size is capable of demonstrating resilience in the face of substantial diet perturbations.

The assembly process and observed diet-induced reconfigurations also proved to be highly reproducible as evidenced by COPRO-Seq results from a replication of E_1_ (experiment 2, “E_2_”). In this follow-up experiment, fecal samples were collected more frequently than in E_1_, providing a dataset with improved temporal resolution. Ordination of E_2_ COPRO-Seq data by PCoA showed that (i) for each treatment group in E_2_, the artificial community assembles in a manner similar to its counterpart in E_1_; (ii) structural reconfigurations in response to diet occur with the same timing as in E_1_; and (iii) the quasi-equilibria achieved during each diet phase are highly similar between experiments for each treatment group (compare Figures 1B and S5A). As in E_1_, cecal data for each E_2_ treatment group overlap with their corresponding fecal samples, and DNA yields from E_2_ fecal samples vary substantially as a function of host diet (Figure S4B).

COPRO-Seq provides precise measurements of the proportional abundance of each member species present in the artificial community. Data collected in both E_1_ and E_2_ (Table S5) revealed significant differences between members in terms of the maximum abundance levels they achieved, the rates at which their abundance levels were impacted by diet shifts, and the degree to which each species demonstrated a preference for one diet over another (Figure S4C). Changes in each species' abundance over time replicated well across animals in each treatment group, suggesting the assembly process and diet-induced reconfigurations occur in an orderly, rules-based fashion and with minimal stochasticity in this artificial community. A species' relative abundance immediately after colonization (i.e., 24 h after gavage/day 1) was, in general, a poor predictor of its abundance at the end of the first diet phase (i.e., day 13) (E_1_ *R* ^2^ = 0.23; E_2_ *R* ^2^ = 0.27), suggesting that early dominance of the founder population was not strongly tied to relative success in the assembly process.

In mice initially fed a HF/HS diet, four *Bacteroides* spp. (*Bacteroides caccae*, *B. cellulosilyticus* WH2, *B. thetaiotaomicron*, and *Bacteroides vulgatus*) each achieved a relative abundance of ≥10% by the end of the first diet phase (day 13 postgavage), with *B. caccae* attaining the highest levels (37.1±4.9% and 34.2±5.5%; group mean ± SD in E_1_ and E_2_, respectively). In animals fed the plant polysaccharide-rich LF/HPP chow during the first diet phase, *B. cellulosilyticus* WH2 was dominant, achieving levels of 37.1±2.0% (E_1_) and 41.6±3.9% (E_2_) by day 13. *B. thetaiotaomicron* and *B. vulgatus* also attained relative abundances of >10%.

Changes in diet often resulted in rapid, dramatic changes in a species' proportional representation. Because the dynamic range of abundance values observed when comparing multiple species was substantial (lowest, *Dorea longicatena* (<0.003%); highest, *B. caccae* (55.0%)), comparing diet responses on a common scale using raw abundance values was challenging. To represent these changes in a way that scaled absolute increases/decreases in relative abundance to the range observed for each strain, we also normalized each species' representation within the artificial community at each time-point to the maximum proportional abundance each microbe achieved across all time-points within each mouse. Plotting the resulting measure of abundance (percentage of maximum achieved; PoMA) over time demonstrates which microbes are strongly responsive to diet (experience significant swings in PoMA value following a diet switch) and which are relatively diet-insensitive (experience only modest or no significant change in PoMA value following a diet switch). Heatmap visualization of E_1_ PoMA values (Figure S5B) indicated that those microbes with a preference for a particular diet in one animal treatment group also tended to demonstrate the same diet preference in the other. Likewise, diet insensitivity was also consistent across treatment groups; diet-insensitive microbes were insensitive regardless of the order in which diets were introduced.

Of the diet-sensitive taxa, those showing the most striking responses were *B. caccae* and *B. ovatus*, which strongly preferred the “Western”-like HF/HS diet and the polysaccharide-rich LF/HPP diet, respectively (Figures 1C and S4C). Among the diet-insensitive taxa, *B. thetaiotaomicron* showed the most stability in its representation (Figures 1C and S4C), consistent with its reputation as a versatile forager. Paradoxically, *B. cellulosilyticus* WH2 was both diet-sensitive and highly fit on its less-preferred diet; although this strain clearly achieved higher levels of representation in animals fed the LF/HPP diet, it also maintained strong levels of representation in animals fed the HF/HS diet (Figures 1C and S4C).

When taking into account the abundance data for all 12 artificial community members, proportional representation at the end of the first diet phase (i.e., day 13) was a good predictor of representation at the end of the third diet phase (i.e., day 42) (E_1_ *R* ^2^ = 0.77; E_2_ *R* ^2^ = 0.84), suggesting that the intervening dietary perturbation had little effect on the ultimate outcomes for most species within this assemblage. However, one very low-abundance strain (*D. longicatena*) achieved significantly different maximum percentage abundances across the two treatment groups in each experiment, suggesting that steady-state levels of this strain may have been impacted by diet history. In mice initially fed the LF/HPP diet, *D. longicatena* was found to persist throughout the experiment at low levels on both diet regimens. In mice initially fed the HF/HS diet, *D. longicatena* dropped below the limit of detection before the end of the first diet phase, was undetectable by the end of the second diet phase, and remained undetectable throughout the rest of the time course. This interesting example raises the possibility that for some species, irreversible hysteresis effects may play a significant role in determining the likelihood that they will persist within a gut over long periods of time.

### The Cecal Metatranscriptome Sampled at the Time of Sacrifice

These diet-induced reconfigurations in the structure of the artificial community led us to examine the degree to which its members were modifying their metabolic strategies. To establish an initial baseline static view of expression data for each microbe on each diet, we developed a custom GeneChip whose probe sets were designed to target 46,851 of the 48,023 known or predicted protein-coding genes within our artificial human gut microbiome (see *Materials and Methods*). Total RNA was collected from the cecal contents of each animal in E_1_ at the time of sacrifice and hybridized to this GeneChip. The total number of genes whose expression was detectable on each diet was remarkably similar (14,929 and 14,594 detected in the LF/HPP→HF/HS→LF/HPP and HF/HS→LF/HPP→HF/HS treatment groups, respectively). A total of 11,373 genes (24.3%) were expressed on both diets (Figure S6A), while 2,003 (4.3%) were differentially expressed to a statistically significant degree, including 161 (6.1%) of the 2,640 genes in the microbiome encoding proteins with CAZy-recognized domains. Figure S6B illustrates the fraction of the community-level CAZome and several species-level CAZomes expressed on each diet (see Table S6 for a comprehensive list of all genes, organized by species and fold-change in expression, whose cecal expression was detectable on each diet and all genes whose expression was significantly different when comparing data from each treatment group).

Among taxa demonstrating obvious diet preferences (as judged by relative abundance data), *B. caccae* and *B. cellulosilyticus* WH2 provided examples of CAZy-level responses to diet change that were different in several respects. Our observations regarding the carbohydrate utilization capabilities and preferences of *B. caccae* are summarized in Text S1. However, our ability to evaluate shifts in *B. caccae*'s metabolic strategy in the gut was limited by its very low abundance in animals fed LF/HPP chow (i.e., our mRNA and subsequent protein assays were often not sensitive enough to exhaustively sample *B. caccae*'s metatranscriptome and metaproteome). In contrast, the abundance of *B. cellulosilyticus* WH2, which favored the LF/HPP diet, remained high enough on both diets to allow for a comprehensive analysis of its expressed genes and proteins. This advantage, along with the exceptional carbohydrate utilization machinery encoded within the genome of this organism, encouraged us to focus on further dissecting the responses of *B. cellulosilyticus* WH2 to diet changes.

**Figure 2:**
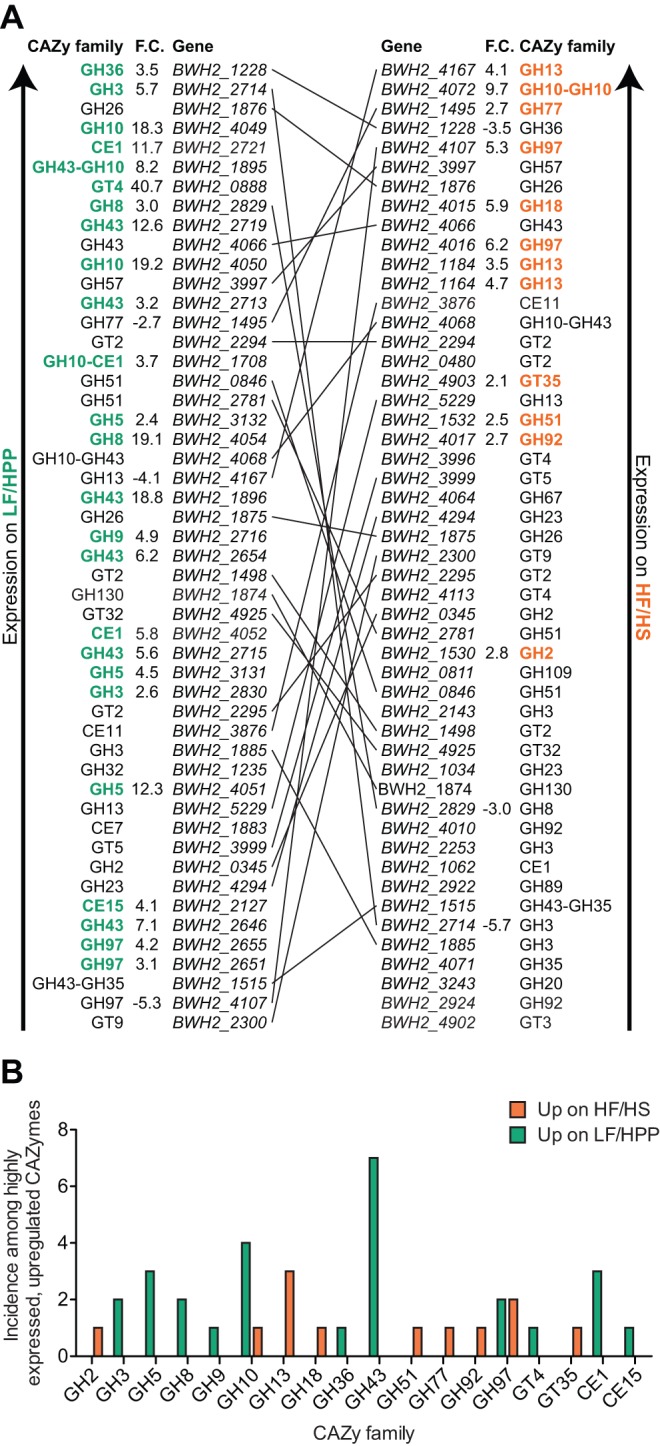
*B. cellulosilyticus* WH2 CAZyme expression in mice fed different diets. (A) Overview of the 50 most highly expressed *B. cellulosilyticus* WH2 CAZymes (GHs, GTs, PLs, and CEs) for samples from each diet treatment group. List position denotes the rank order of gene expression for each treatment group, with higher expression levels situated at the top of each list. Genes common to both lists are identified by a connecting line, with the slope of the line indicating the degree to which a CAZyme's prioritized expression is increased/decreased from one diet to the other. CAZy families in bold, colored letters highlight those list entries found to be significantly up-regulated relative to the alternative diet (i.e., a CAZyme with a bold green family designation was up-regulated on the LF/HPP diet; a bold orange family name implies a gene was up-regulated significantly on the HF/HS diet). Statistically significant fold-changes between diets are denoted in the “F.C.” column (nonsignificant fold-changes are omitted for clarity). (B) Breakdown by CAZy family of the top 10% most expressed CAZymes on each diet whose expression was also found to be significantly higher on one diet than the other. Note that for each diet, the family with the greatest number of up-regulated genes was also exclusively up-regulated on that diet (LF/HPP, GH43; HF/HS, GH13). In total, 25 genes representative of 27 families and 12 genes representative of 13 families are shown for the LF/HPP and HF/HS diets, respectively.

Detailed inspection of the expressed *B. cellulosilyticus* WH2 CAZome (503 CAZymes in total) provided an initial view of this microbe's sophisticated carbohydrate utilization strategy. A comparison of the top decile of expressed CAZymes on each diet disclosed many shared elements between the two lists, spanning many different CAZy families, with just over half of the 50 most expressed enzymes on the plant polysaccharide-rich LF/HPP chow also occurring in the list of most highly expressed enzymes on the sucrose-, corn starch-, and maltodextrin-rich HF/HS diet (Figure 2A). Twenty-five of the 50 most expressed CAZymes on the LF/HPP diet were significantly up-regulated compared to the HF/HS diet; of these, seven were members of the GH43 family (Figure 2B). The GH43 family consists of enzymes with activities required for the breakdown of plant-derived polysaccharides such as hemicellulose and pectin. Inspection of the enzyme commission (EC) annotations for the most up-regulated GH43 genes shows that they encode xylan 1,4-β-xylosidases (EC 3.2.1.37), arabinan endo-1,5-α-L-arabinosidases (EC 3.2.1.99), and α-L-arabinofuranosidases (EC 3.2.1.55). The GH10 family, which is currently comprised exclusively of endo-xylanases (EC 3.2.1.8, EC 3.2.1.32), was also well represented among this set of 25 genes, with four of the seven putative GH10 genes in the *B. cellulosilyticus* WH2 genome making the list. Strikingly, of the 45 predicted genes with putative GH43 domains in the *B. cellulosilyticus* WH2 genome, none were up-regulated on the “Western”-style HF/HS diet.

The most highly expressed *B. cellulosilyticus* WH2 CAZyme on the plant polysaccharide-rich chow (which was also highly-expressed on the HF/HS chow) was BWH2_1228, a putative α-galactosidase from the GH36 family. These enzymes, which are not expressed by humans in the stomach or intestine, cleave terminal galactose residues from the nonreducing ends of raffinose family oligosaccharides (RFOs, including raffinose, stachyose, and verbascose), galacto(gluco)mannans, galactolipids, and glycoproteins. RFOs, which are well represented in cereal grains consumed by humans, are expected to be abundant in the LF/HPP diet given its ingredients (e.g., soybean meal), but potential substrates in the HF/HS diet are less obvious, possibly implicating a host glycolipid or glycoprotein target.

Surface glycans in the intestinal epithelium of rodents are decorated with terminal fucose residues [34] as well as terminal sialic acid and sulfate [35]. Hydrolysis of the α-2 linkage connecting terminal fucose residues to the galactose-rich extended core is thought to be catalyzed in large part by GH95 and GH29 enzymes [36]. The *B. cellulosilyticus* WH2 genome is replete with putative GH95 and GH29 genes (total of 12 and 9, respectively), but only a few (*BWH2_1350*/*2142*/*3154*/*3818*) were expressed *in vivo* on at least one diet, and their expression was low relative to many other CAZymes (see Table S6). Cleavage of terminal sialic acids present in host mucins by bacteria is usually carried out by GH33 family enzymes. *B. cellulosilyticus* WH2 has two GH33 genes that are expressed on either one diet (*BWH2_3822*, HF/HS) or both diets (*BWH2_4650*), but neither is highly expressed relative to other *B. cellulosilyticus* WH2 CAZymes. Therefore, utilization of host glycans by *B. cellulosilyticus* WH2, if it occurs, likely requires partnerships with other members of the artificial community that express GH29/95/33 enzymes (see Table S6 for a list of members that express these enzymes in a diet-independent and/or diet-specific fashion).

Among the 50 most highly expressed *B. cellulosilyticus* WH2 CAZymes, 12 were significantly up-regulated on the HF/HS diet compared to the LF/HPP diet, with members of family GH13 being most prevalent. While the enzymatic activities and substrate specificities of GH13 family members are varied, most relate to the hydrolysis of substrates comprising chains of glucose subunits, including amylose (one of the two components of starch) and maltodextrin, both prominent ingredients in the HF/HS diet.

GeneChip-based profiling of the E_1_ cecal communities provided a snapshot of the metatranscriptome on the final day of the final diet phase in each treatment group. The analysis of *B. cellulosilyticus* WH2 CAZyme expression suggested that this strain achieves a “generalist” lifestyle not by relying on substrates that are present at all times (e.g., host mucins), but rather by modifying its resource utilization strategy to effectively compete with other microbes for diet-derived polysaccharides that are not metabolized by the host.

### Community-Level Analysis of Diet-Induced Changes in Microbial Gene Expression

To develop a more complete understanding of the dynamic changes that occur in gene expression over time and throughout the artificial community following diet perturbations, we performed microbial RNA-Seq analyses using feces obtained at select time-points from mice in the LF/HPP→HF/HS→LF/HPP treatment group of E_2_ (Figure S3).

**Figure 3:**
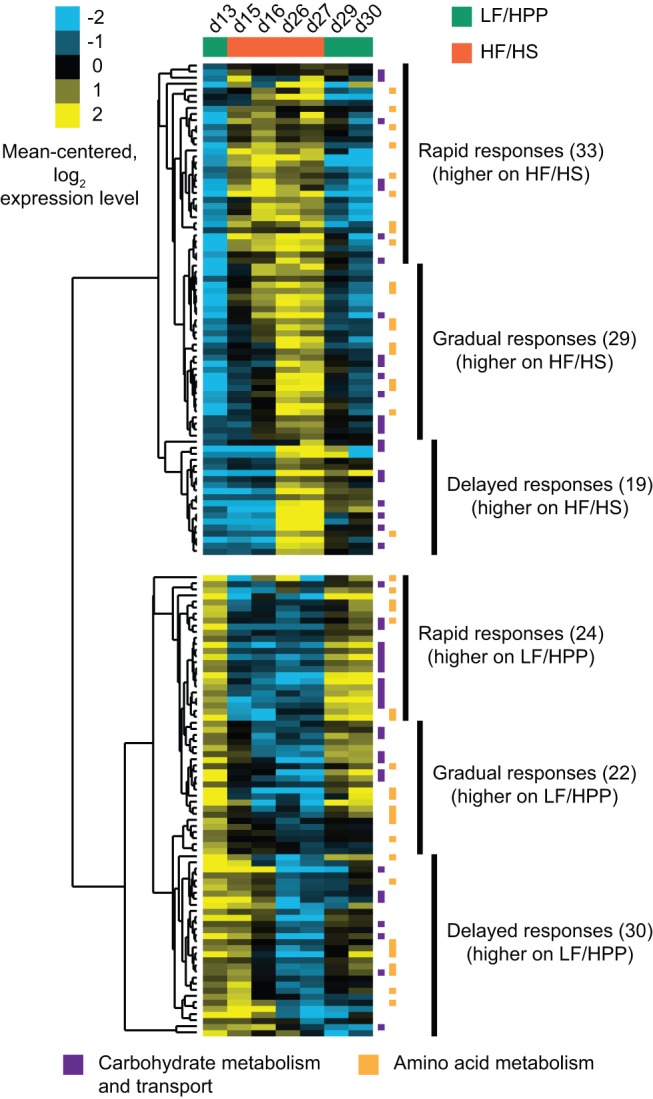
Top-down analysis of fecal microbiome RNA expression in mice receiving oscillating diets. The fecal metatranscriptomes of four animals in the LF/HPP→HF/HS→LF/HPP treatment group of E_2_ were analyzed using microbial RNA-Seq at seven time-points to evaluate the temporal progression of changes in expressed microbial community functions triggered by a change in diet. After aligning reads to genes in the defined artificial human gut microbiome, raw counts were collapsed by the functional annotation (EC number) of the gene from which the corresponding reads originated. Total counts for each EC number in each sample were normalized, and any EC numbers demonstrating a statistically significant difference in their representation in the metatranscriptome between the final days of the first two diet phases were identified using a model based on the negative binomial distribution [57]. Normalized expression values for 157 significant EC numbers (out of 1,021 total tested) were log-transformed, mean-centered, and subjected to hierarchical clustering, followed by heatmap visualization. “Rapid” responses are those where expression increased/decreased dramatically within 1–2 d of a diet switch. “Gradual” responses are those where expression changed notably, but slowly, between the two diet transition points. “Delayed” responses are those where significant expression changes did not occur until the end of a diet phase. EC numbers specifying enzymatic reactions relevant to carbohydrate metabolism and/or transport are denoted by purple markers, while those with relevance to amino acid metabolism are indicated using orange markers. A full breakdown of all significant responses over time and the outputs of the statistical tests performed are provided in Table S7.

We began with a “top-down” analysis in which every RNA-Seq read count from every gene in the artificial microbiome was binned based on the functional annotation of the gene from which it was derived, regardless of its species of origin. In this case, the functional annotation used as the binning variable was the predicted EC number for a gene's encoded protein product. Expecting that some changes might occur rapidly, while others might require days or weeks, we searched for significant differences between the terminal time-points of the first two diet phases (i.e., points at which the model human gut microbiota had been allowed 13 d to acclimate to each diet). The 157 significant changes we identified were subjected to hierarchical clustering by EC number to determine which functional responses occurred with similar kinetics. The results revealed that in contrast to the rapid, diet-induced structural reconfigurations observed in this artificial community, community-level changes in microbial gene expression occurred with highly variable timing that differed from function to function. These changes were dominated by EC numbers associated with enzymatic reactions relevant to carbohydrate and amino acid metabolism (see Table S7 for a summary of all significant changes observed, including aggregate expression values for each functional bin (EC number) at each time-point). Significant responses could be divided into one of three groups: “rapid” responses were those where the representation of EC numbers in the transcriptome increased/decreased dramatically within 1–2 d of a diet switch; “gradual” responses were those where the representation of EC numbers changed notably, but slowly, between the two diet transition points; and “delayed” responses were those where significant change did not occur until the end of a diet phase (Figure 3, Table S7). EC numbers associated with reactions important in carbohydrate metabolism and transport were distributed across all three of these response types for each of the two diets. Nearly all genes encoding proteins with EC numbers related to amino acid metabolism that were significantly up-regulated on HF/HS chow binned into the “rapid” or “gradual” groups, suggesting this diet put immediate pressure on the artificial microbial community to increase its repertoire of expressed amino acid biosynthesis and degradation genes. Genes with assigned EC numbers involved in amino acid metabolism that were significantly up-regulated on the other, polysaccharide-rich, LF/HPP diet were spread more evenly across these three response types (Figure 3).

Careful inspection of our top-down analysis results and a complementary “bottom-up” analysis in which normalization was performed at the level of individual species, rather than at the community level, allowed us to identify other important responses that would have gone undetected were it not for the fact that we were dealing with a defined assemblage of microbes where all of the genes in component members' genomes were known. For example, an assessment of the representation of EC 3.2.1.8 (endo-1,4-β-xylanase) within the metatranscriptome before and after the first diet switch (LF/HPP→HF/HS) initially suggested that this activity was reduced to a statistically significant degree as a result of the first diet perturbation (day 13 versus day 27; Mann–Whitney *U* test, *p* = 0.03; Figure S7A). Aggregation by species of all sequencing read counts assignable to mRNAs encoding proteins with this EC number revealed that over 99% of the contributions to this functional bin originated from *B. cellulosilyticus* WH2 (note the similarity in a comparison of Figure S7A and Figure S7B), implying that the community-level response and the response of this *Bacteroides* species were virtually one and the same. A tally of all sequencing reads assignable to *B. cellulosilyticus* WH2 at each time-point disclosed that although this strain maintains high proportional representation in the artificial community throughout each diet oscillation period (range, 10.3–42.5% and 11.6–43.3% for E_1_ and E_2_, respectively), its contribution to the metatranscriptome is substantially decreased during the HF/HS diet phase (Figure S7C). This dramatic reduction in the extent to which *B. cellulosilyticus* WH2 contributes to the metatranscriptome in HF/HS-fed mice “masks” the significant up-regulation of EC 3.2.1.8 that occurs within the *B. cellulosilyticus* WH2 transcriptome following the first diet shift (day 13 versus day 27; Mann–Whitney *U* test, *p* = 0.03; Figure S7D). A further breakdown of endo-1,4-β-xylanase up-regulation in *B. cellulosilyticus* WH2 when mice are switched to the HF/HS diet reveals that most of this response is driven by two genes, *BWH2_4068* and *BWH2_4072* (Figure S7E). Our realization that we were unable to correctly infer the direction of one of the most significant diet-induced gene expression changes in the second most abundant strain in the artificial community when inspecting functional responses at the community level provides a strong argument for expanding the use of microbial assemblages comprised exclusively of sequenced species in studies of the gut microbiota. This should allow the contributions of individual species to community activity to be evaluated in a rigorous way that is not possible with microbial communities of unknown or poorly defined gene composition.

### High-Resolution Profiling of the Cecal Metaproteome Sampled at the Time of Sacrifice

In principle, protein measurements can provide a more direct readout of expressed community functions than an RNA-level analysis, and thus a deeper understanding of community members' interactions with one another and with their habitat [37],[38]. For these reasons and others, much work has been dedicated to applying shotgun proteomics techniques to microbial ecosystems in various environments [39],[40]. Though these efforts have provided illustrations of significant methodological advances, they have been limited by the complexity of the metaproteomes studied and by the difficulties this complexity creates when attempting to assign peptide identities uniquely to proteins of specific taxa. Recognizing that a metaproteomics analysis of our artificial community would not be subject to such uncertainty given its fully defined microbiome and thus fully defined theoretical proteome, we subjected cecal samples from two mice from each diet treatment group in E_1_ (*n* = 4 total) to high-performance liquid chromatography-tandem mass spectrometry (LC-MS/MS; see *Materials and Methods*). We had three goals: (i) to evaluate how our ability to assign peptide-spectrum matches (PSMs) to particular proteins within a theoretical metaproteome is affected by the presence of close homologs within the same species and within other, closely related species; (ii) to test the limits of our ability to characterize protein expression across different species given the substantial dynamic range we documented in microbial species abundance; and (iii) to collect semiquantitative peptide/protein data that might validate and enrich our understanding of functional responses identified at the mRNA level, particularly with respect to the niche (profession) of CAZyme-rich *B. cellulosilyticus* WH2.

Given the evolutionary relatedness of the strains involved, we expected that some fraction of observed PSMs from each sample would be of ambiguous origin due to nonunique peptides shared between species' proteomes. To assess which species might be most affected by this phenomenon when characterizing the metaproteome on different diets, we catalogued each strain's theoretical peptidome using an *in silico* tryptic digest. This simulated digest took into account both the potential for missed trypic cleavages and the peptide mass range that could be detected using our methods. The results (Figure S8A, Table S8) demonstrated that for an artificial community of modest complexity, the proportion of peptides within each strain's theoretical peptidome that are “unique” (i.e., assignable to a single protein within the theoretical metaproteome) varies substantially from species to species, even among those that are closely related. We found the lone representative of the *Actinobacteria* in the artificial community, *Collinsella aerofaciens*, to have the highest proportion of unique peptides (94.2%), while *B. caccae* had the lowest (63.0%). Interestingly, there was not a strong correlation between the fraction of a species' peptides that were unique and the total number of unique peptides that species contributed to the theoretical peptidome. For example, *C. aerofaciens* (2,367 predicted protein-coding genes) contributed only 81,894 (1.5%) unique peptides, the lowest of any artificial community member evaluated, despite having a proteome composed of mostly unique peptides. On the other hand, *B. cellulosilyticus* WH2 (5,244 predicted protein-coding genes) contributed 241,473 (4.5%) unique peptides, the highest of any member despite a high fraction of nonunique peptides (18.4%) within its theoretical peptidome. The evolutionary relatedness of the *Bacteroides* components of the artificial community appeared to negatively affect our ability to assign their peptides to specific proteins; their six theoretical peptidomes had the six lowest uniqueness levels. However, their greater number of proteins and peptides relative to the *Firmicutes* and *Actinobacteria* more than compensated for this deficiency; over 60% of unique peptides within the unique theoretical metaproteome were contributed by the *Bacteroides*.

We also found that the proportion of PSMs uniquely assignable to a single protein within the metaproteome varied significantly by function, suggesting that some classes of proteins can be traced back to specific microbes more readily than others. For example, when considering all theoretical peptides that could be derived from the proteome of a particular bacterial species, those from proteins with roles in categories with high expected levels of functional conservation (e.g., translation and nucleotide metabolism) were on average deemed unique more often than those from proteins with roles in functions we might expect to be less conserved (e.g., glycan biosynthesis and metabolism) (see Table S8 for a summary of how peptide uniqueness varied across different KEGG categories and pathways, and across different species in the experiment). However, even in KEGG categories and pathways with high expected levels of functional conservation, the vast majority of peptides were found to be unique when a particular species was not closely related to other members of the artificial community.

Next, we determined the average number of proteins that could be experimentally identified in our samples for each microbial species within each treatment group in E_1_. The results (Figure S8B, Table S9) illustrate two important conclusions. First, although equal concentrations of total protein were evaluated for each sample, slightly less than twice as many total microbial proteins were identified in samples from the LF/HPP-fed mice as those from mice fed the HF/HS diet (4,659 versus 2,777, respectively). While there are a number of possible explanations, both this finding and the higher number of mouse proteins detected in samples from HF/HS-fed animals are consistent with the results of our fecal DNA analysis, which indicated that the HF/HS diet supports lower levels of gut microbial biomass than the LF/HPP diet (Figure S4A,B). Second, a breakdown of all detected microbial proteins by species of origin (Figure S8B) revealed that the degree to which we could inspect protein expression for a given species was dictated largely by its relative abundance and the diet to which it was exposed.

Our ability to detect many of *B. cellulosilyticus* WH2's expressed transcripts and proteins in samples from both diet treatment groups allowed us to determine how well RNA and protein data for an abundant, active member of the artificial community might correlate. These data also allowed us to evaluate whether or not the types of genes considered might influence the degree of correlation between these two datasets. We first performed a spectral count-based correlation analysis on the diet-induced, log-transformed, average fold-differences in expression for all *B. cellulosilyticus* WH2 genes that were detectable at both the RNA and protein level for both diets. The results revealed a moderate degree of linear correlation between RNA and protein observations (Figure S8C, black plot; *r* = 0.53). However, subsequent division of these genes into functionally related subsets, which were each subjected to their own correlation analysis, revealed striking differences in the degree to which RNA-level and protein-level expression changes agreed with one another. For example, diet-induced changes in mRNA expression for genes involved in translation showed virtually no correlation with changes measured at the protein level (Figure S8C, red plot; *r* = 0.03). Correlations for other categories of *B. cellulosilyticus* WH2 genes, such as those involved in energy metabolism (Figure S8C, green plot; *r* = 0.36) and amino acid metabolism (Figure S8C, orange plot; *r* = 0.48), were also poorer than the correlation for the complete set of detectable genes. In contrast, the correlation for the 110 genes with predicted involvement in carbohydrate metabolism was quite strong (Figure S8C, blue plot; *r* = 0.69), and was in fact the best correlation identified for any functional category of genes considered. The significant range of correlations observed in different categories of genes suggests that the degree to which RNA-based analyses provide an accurate picture of microbial adaptation to environmental perturbation may be strongly impacted by the functional classification of the genes involved. Additionally, these data further emphasize the need for enhanced dynamic range metaproteome measurements and better bioinformatic methods to assign/bin unique and nonunique peptides so that deeper and more thorough surveys of the microbial protein landscape can be performed and evaluated alongside more robust transcriptional datasets.

### Identifying Two Diet-Inducible, Xylanase-Containing PULs Whose Genetic Disruption Results in Diet-Specific Loss of Fitness

**Figure 4:**
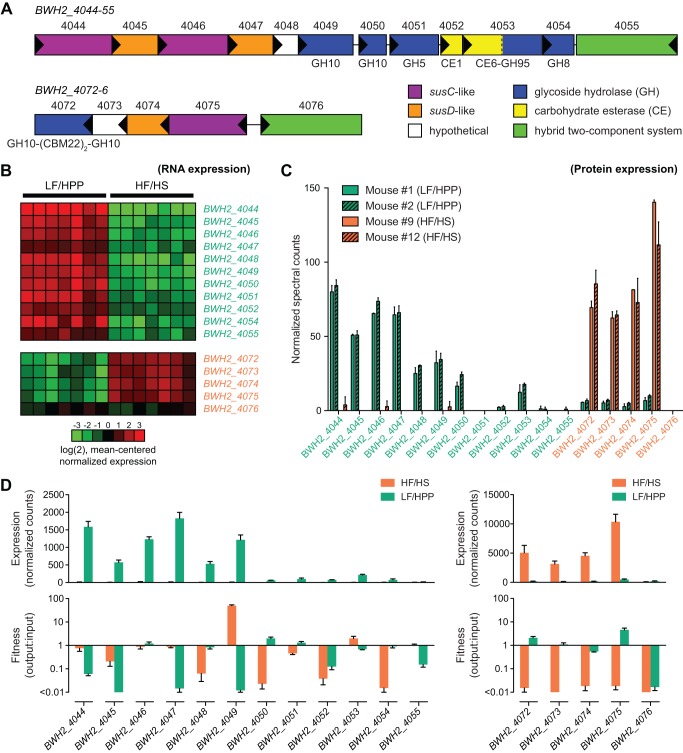
Two xylanase-containing *B. cellulosilyticus* WH2 PULs demonstrating strong diet-specific expression patterns *in vivo*. (A) The PUL spanning *BWH2_4044*–*55* includes a four-gene cassette comprising two consecutive *susC*/*D* pairs, multiple genes encoding GHs and CEs, and a gene encoding a putative hybrid two-component system (HTCS) presumed to play a role in the regulation of this locus. GH10 enzymes are endo-xylanases (most often endo-β-1,4-xylanases), while some GH5 and GH8 enzymes are also known to have endo- or exo-xylanase activity. CE6 enzymes are acetyl xylan esterases, as are some members of the CE1 family. A second PUL spanning *BWH2_4072*–*6* contains a *susC*/*D* cassette, an endo-xylanase with dual GH10 modules as well as dual carbohydrate (xylan) binding modules (CBM22), a hypothetical protein of unknown function, and a putative HTCS. (B) Heatmap visualization of GeneChip expression data for *BWH2_4044*–*55* and *BWH2_4072*–*6* showing marked up-regulation of these putative PULs when mice are fed either a plant polysaccharide-rich LF/HPP diet or a diet high in fat and simple sugar (HF/HS), respectively. Data are from cecal contents harvested from mice at the endpoint of experiment E_1_. (C) Mass spectrometry-based quantitation of the abundance of all cecal proteins from the *BWH2_4044*–*55* and *BWH2_4072*–*6* PULs that were detectable in the same material used for GeneChip quantitation in panel (B). Bars represent results (mean ± SEM) from two technical runs per sample. For each MS run, the spectral counts for each protein were normalized against the total number of *B. cellulosilyticus* WH2 spectra acquired. (D) Comparison of *in vivo* PUL gene expression as measured by RNA-Seq (top) and the degree to which disruption of each gene within each PUL by a transposon impacts the fitness of *B. cellulosilyticus* WH2 on each diet, as measured by insertion sequencing (INSeq, bottom). For the lower plots, fitness measurements were calculated by dividing a mutant's representation (normalized sequencing counts) within the fecal output population by its representation within an input population that was introduced into germ-free animals via a single oral gavage along with other members of the artificial community. For cases in which no instances of a particular mutant could be measured in the fecal output (resulting in a fitness calculation denominator of zero), data are plotted as “<0.01” and are drawn without error bars.

Several of the most highly expressed and diet-sensitive *B. cellulosilyticus* WH2 genes in this study fell within two putative PULs. One PUL (*BWH2_4044–55*) contains 12 ORFs that include a dual *susC*/*D* cassette, three putative xylanases assigned to CAZy families GH8 and GH10, a putative multifunctional acetyl xylan esterase/α-L-fucosidase, and a putative hybrid two-component system regulator (Figure 4A). Gene expression within this PUL was markedly higher in mice consuming the plant polysaccharide-rich LF/HPP diet at both the mRNA and protein level. Our mRNA-level analysis disclosed that *BWH2_4047* was the most highly expressed *B. cellulosilyticus* WH2 *susD* homolog on this diet. Likewise, *BWH2_4046*/*4*, the two *susC*-like genes within this PUL, were the 2nd and 4th most highly expressed *B. cellulosilyticus* WH2 *susC*-like genes in LF/HPP-fed animals, and exhibited expression level reductions of 99.5% and 93% in animals consuming the HF/HS diet. The same LF/HPP diet bias was observed for other genes within this PUL (Figures 2A and 4B) but not for neighboring genes. The same trends were obvious and amplified when we quantified protein expression (Figure 4C). In mice fed LF/HPP chow, only three *B. cellulosilyticus* WH2 SusC-like proteins had higher protein levels than BWH2_4044/6, and only two SusD-like proteins had higher levels than BWH2_4045/7. Strikingly, we were unable to detect a single peptide from 9 of the 12 proteins in this PUL in samples obtained from mice fed the HF/HS diet, emphasizing the strong diet specificity of this locus.

A second PUL in the *B. cellulosilyticus* WH2 genome composed of a *susC*/*D*-like pair (*BWH2_4074*/*5*), a putative hybrid two-component system regulator (*BWH2_4076*), and a xylanase (GH10) with dual carbohydrate binding module domains (CBM22) (*BWH2_4072*) (Figure 4A) demonstrated a strong but opposite diet bias, in this case exhibiting significantly higher expression in animals consuming the HF/HS “Western”-like diet. Our mRNA-level analysis showed that this xylanase was the second most highly expressed *B. cellulosilyticus* WH2 CAZyme in animals consuming this diet (Figure 2A). As with the previously described PUL, shotgun metaproteomics validated the transcriptional analysis (Figure 4B,C); with the exception of the gene encoding the PUL's presumed transcriptional regulator (*BWH2_4076*), diet specificity was substantial, with protein-level fold changes ranging from 10–33 across the locus (Table S10).

Recent work by Cann and co-workers has done much to advance our understanding of the regulation and metabolic role of xylan utilization system gene clusters in xylanolytic members of the Bacteroidetes, particularly within the genus *Prevotella* [41]. The “core” gene cluster of the prototypical xylan utilization system they described consists of two tandem repeats of *susC*/*susD* homologs (*xusA*/*B*/*C*/*D*), a downstream hypothetical gene (*xusE*) and a GH10 endoxylanase (*xyn10C*). The 12-gene PUL identified in our study (*BWH2_4044*–*55*) appears to contain the only instance of this core gene cluster within the *B. cellulosilyticus* WH2 genome, suggesting that this PUL, induced during consumption of a plant polysaccharide-rich diet, is likely to be the primary xylan utilization system within this organism. A recent study characterizing the carbohydrate utilization capabilities of *B. ovatus* ATCC 8483 also identified two PULs involved in xylan utilization (*BACOVA_04385*–*94*, *BACOVA_03417*–*50*) whose gene configurations differ from those described in *Prevotella* spp. [25]. Interestingly, the five proteins encoded by the smaller xylanase-containing PUL described above (*BWH2_4072*–*6*) are homologous to the products of the last five genes in *BACOVA_4385*–*94* (i.e., *BACOVA_4390*–*4*). The order of these five genes in these two loci is also identical. The similarities and differences observed when comparing the putative xylan utilization systems encoded within the genomes of different Bacteroidetes illustrate how its members may have evolved differentiated strategies for utilizing hemicelluloses like xylan.

Having established that expression of *BWH2_4044*–*55* and *BWH2_4072*–*6* is strongly dictated by diet, we next sought to determine if these PULs are required by *B. cellulosilyticus* WH2 for fitness *in vivo*. A follow-up study was performed in which mice were fed either a LF/HPP or HF/HS diet after being colonized with an artificial community similar to the one used in E_1_ and E_2_ (see *Materials and Methods*). The wild-type *B. cellulosilyticus* WH2 strain used in our previous experiments was replaced with a transposon mutant library consisting of over 90,000 distinct transposon insertion mutants in 91.5% of all predicted ORFs (average of 13.9 distinct insertion mutants per ORF). The library was constructed using methods similar to those reported by Goodman et al. ([42]; see *Materials and Methods*) so that the relative proportion of each insertion mutant in both the input (orally gavaged) and output (fecal) populations could be determined using insertion sequencing (INSeq). The INSeq results revealed clear, diet-specific losses of fitness when components of these loci were disrupted (Figure 4D). Additionally, as observed in E_1_ and E_2_, expression of each PUL was strongly biased by diet, with genes *BWH2_4072*–*5* demonstrating up-regulation on the HF/HS diet and *BWH2_4044*–*55* on the LF/HPP diet. The extent to which a gene's disruption impacted the fitness of *B. cellulosilyticus* WH2 on one diet or the other correlated well with whether or not that gene was highly expressed on a given diet. For example, four of the five most highly expressed genes in the *BWH2_4044*–*55* locus were the four genes shown to be most crucial for fitness on the LF/HPP diet. Of these four genes, three were *susC* or *susD* homologs (the fourth was the putative endo-1,4-β-xylanase thought to constitute the last element of the xylan utilization system core). Though the fitness cost of disrupting genes within *BWH2_4044*–*55* varied from gene to gene, disruption of any one component of the *BWH2_4072*–*6* PUL had serious consequences for *B. cellulosilyticus* WH2 in animals fed the HF/HS diet. This could suggest that while disruption of some components of the *BWH2_4044*–*55* locus can be rescued by similar or redundant functions elsewhere in the genome, the same is not true for *BWH2_4072*–*5*. Notably, disruption of *BWH2_4076*, which is predicted to encode a hybrid two-component regulatory system, had negative consequences on either diet tested, indicating that regulation of this locus is crucial even when the PUL is not actively expressed. While many genes outside of these two PULs were also found to be important for the *in vivo* fitness of *B. cellulosilyticus* WH2, those within these PULs were among the most essential to diet-specific fitness, suggesting that these loci are central to the metabolic lifestyle of *B. cellulosilyticus* WH2 in the gut.

### Characterizing the Carbohydrate Utilization Capabilities of *B. cellulosilyticus* WH2 and *B. caccae*

The results described in the preceding section indicate that *B. cellulosilyticus* WH2 prioritizes xylan as a nutrient source in the gut and that it tightly regulates the expression of its xylan utilization machinery. Moreover, the extraordinary number of putative CAZymes and PULs within the *B. cellulosilyticus* WH2 genome suggests that it is capable of growing on carbohydrates with diverse structures and varying degrees of polymerization. To characterize its carbohydrate utilization capabilities, we defined its growth in minimal medium (MM) supplemented with one of 46 different carbohydrates [25]. Three independent growths, each consisting of two technical replications, yielded a total of six growth curves for each substrate. Of the 46 substrates tested, *B. cellulosilyticus* WH2 grew on 39 (Table S11); they encompassed numerous pectins (6 of 6), hemicelluloses/β-glucans (8 of 8), starches/fructans/α-glucans (6 of 6), and simple sugars (14 of 15), as well as host-derived glycans (4 of 7) and one cellooligosaccharide (cellobiose). The seven substrates that did not support growth included three esoteric carbohydrates (carrageenan, porphyran, and alginic acid), the simple sugar N-acetylneuraminic acid, two host glyans (keratan sulfate and mucin O-glycans), and fungal cell wall-derived α-mannan. *B. cellulosilyticus* WH2 clearly grew more robustly on some carbohydrates than others. Excluding simple sugars, fastest growth was achieved on dextran (0.099±0.048 OD_600_ units/h), laminarin (0.095±0.014), pectic galactan (0.088±0.018), pullulan (0.088±0.026), and amylopectin (0.085±0.003). Although one study has reported that the type strain of *B. cellulosilyticus* degrades cellulose [43], the WH2 strain failed to demonstrate any growth on MM plus cellulose (specifically, Solka-Floc 200 FCC from International Fiber Corp.) after 5 d. Maximum cell density was achieved with amylopectin (1.17±0.02 OD_600_ units), dextran (1.12±0.20), cellobiose (1.09±0.08), laminarin (1.08±0.08), and xyloglucan (0.99±0.04). Total *B. cellulosilyticus* WH2 growth (i.e., maximum cell density achieved) on host-derived glycans was typically very poor, with only two substrates achieving total growth above 0.2 OD_600_ units (chondroitin sulfate, 0.50±0.04; glycogen, 0.99±0.02). The disparity between total growth on plant polysaccharides versus host-derived glycans, including O-glycans that are prevalent in host mucin, indicates a preference for diet-derived saccharides, consistent with our *in vivo* mRNA and protein expression data.

We also determined how the growth rate of *B. cellulosilyticus* WH2 on these substrates compared to the growth rates for other prominent gut *Bacteroides* spp. After subjecting *B. caccae* to the same phenotypic characterization as *B. cellulosilyticus* WH2, we combined our measurements for these two strains with previously published measurements for *B. thetaiotaomicron* and *B. ovatus* [25]. The results underscored the competitive growth advantage *B. cellulosilyticus* WH2 likely enjoys when foraging for polysaccharides in the intestinal lumen. For example, of the eight hemicelluloses and β-glucans tested in our carbohydrate panel, *B. cellulosilyticus* WH2 grew fastest on six while *B. ovatus* grew fastest on two (Table S11). *B. caccae* and *B. thetaiotaomicron*, on the other hand, failed to grow on any of these substrates. Across all the carbohydrates for which data are available for all four species, *B. cellulosilyticus* WH2 grew fastest on the greatest number of polysaccharides (11 of 26) and tied with *B. caccae* for the greatest number of monosaccharides (6 of 15). *B. thetaiotaomicron* and *B. caccae* did, however, outperform the other two *Bacteroides* tested with respect to utilization of host glycans *in vitro*, demonstrating superior growth rates on four of five substrates tested (Table S11).

**Figure 5:**
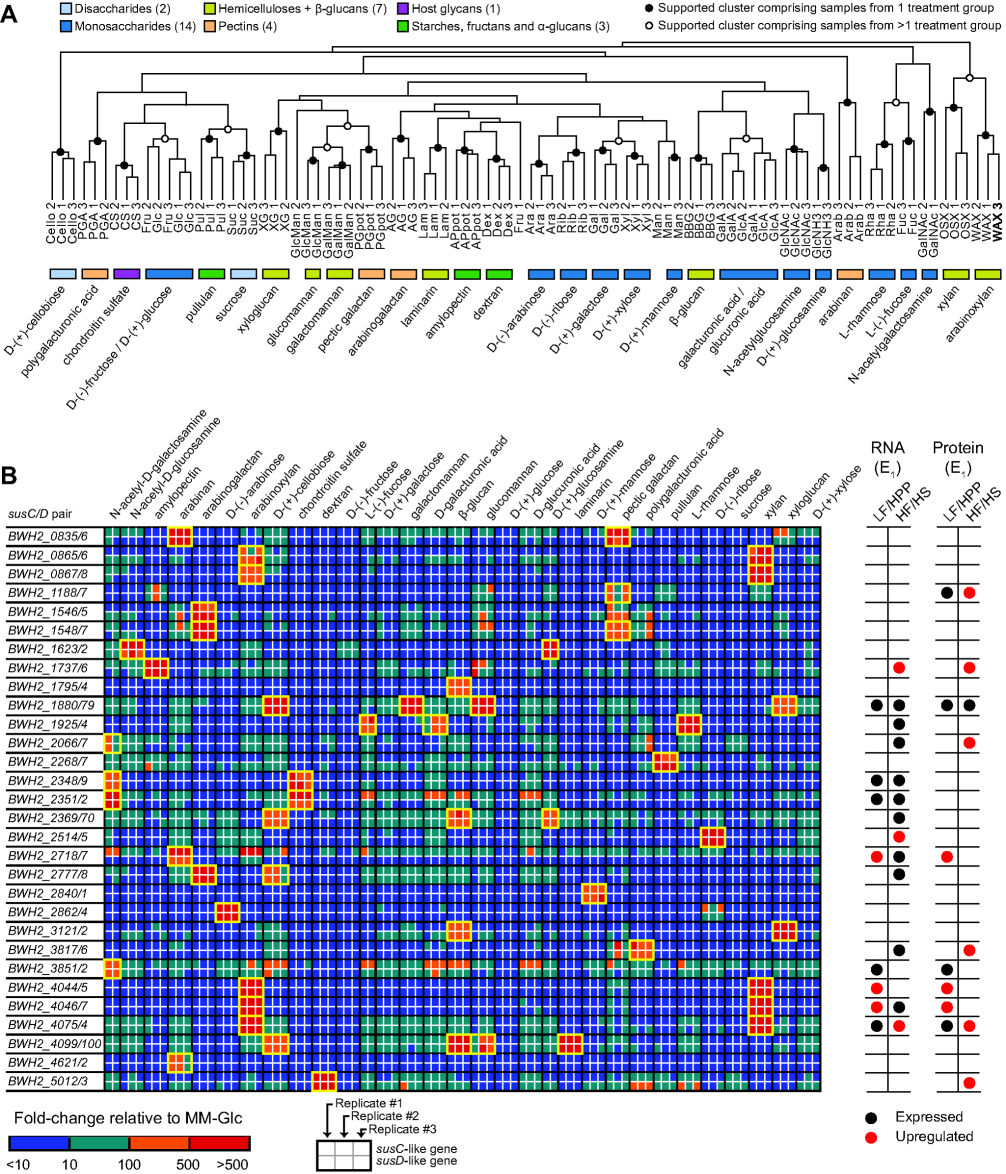
*In vitro* microbial RNA-Seq profiling of *B. cellulosilyticus* WH2 during growth on different carbohydrates. (A) Hierarchical clustering of the gene expression profiles of 90 cultures grown in minimal medium supplemented with one of 31 simple or complex sugars (*n* = 2–3 replicates per condition). Circles at dendrogram branch points identify clusters with strong bootstrapping support (>95%; 10,000 repetitions). Solid circles denote clusters comprising only replicates from a single treatment group/carbohydrate, while open circles denote higher level clusters comprising samples from multiple treatment groups. Colored rectangles indicate the type of carbohydrate on which the samples within each cluster were grown. (B) Unclustered heatmap representation of fold-changes in gene expression relative to growth on minimal medium plus glucose (MM-Glc) for 60 of the 236 paired *susC*- and *susD*-like genes identified within the *B. cellulosilyticus* WH2 genome (for a full list of all paired and unpaired *susC* and *susD* homologs, see Table S2). Data shown are limited to those genes whose expression on at least one of the 31 carbohydrates tested demonstrated a >100-fold increase relative to growth on MM-Glc for at least one of the replicates within the treatment group. Yellow boxes denote areas of the map where both genes in a *susC*/*D* pair were up-regulated >100-fold for at least two of the replicates in a treatment group and where the average up-regulation for each gene in the pair was >100-fold across all replicates of the treatment group. Two sets of columns to the right of the heatmap indicate PULs that were detectably expressed at the mRNA level (left set of columns) and/or protein level (right set of columns) in experiment 1 (E_1_). Red and black circles indicate that both genes in a *susC*/*D* pair were consistently expressed on a particular diet, as determined by GeneChip analysis of cecal RNA (≥5 of 7 animals assayed) or LC-MS/MS analysis of cecal protein (2 of 2 animals assayed). In both cases, a red circle denotes significantly higher expression on one diet compared to the other.

*B. cellulosilyticus* WH2's rapid growth to high densities on xylan, arabinoxylan, and xyloglucan, as well as xylose, arabinose, and galactose, is noteworthy given our prediction that two of its most tightly regulated, highly expressed PULs appear to be involved in the utilization of xylan, arabinoxylan, or some closely related polysaccharide. To identify specific mono- and/or polysaccharides capable of triggering the activation of these two PULs, as well as the 111 other putative PULs within the *B. cellulosilyticus* WH2 genome, we used RNA-Seq to characterize its transcriptional profile at mid-log phase in MM (Table S12) plus one of 16 simple sugars or one of 15 complex sugars (Table S13) (see *Materials and Methods*; *n* = 2–3 cultures/substrate; 5.2–14.0 million raw Illumina HiSeq reads generated for each of the 90 transcriptomes). After mapping each read to the *B. cellulosilyticus* WH2 reference gene set, counts were normalized using DESeq to allow for direct comparisons across samples and conditions. Hierarchical clustering of the normalized dataset resulted in a well-ordered dendrogram in which samples clustered almost perfectly by the carbohydrate on which *B. cellulosilyticus* WH2 was grown (Figure 5A). The consistency of this clustering illustrates that (i) technical replicates within each condition exhibit strong correlations with one another, suggesting any differences between cultures in a treatment group (e.g., small differences in density or growth phase) had at best minor effects on aggregate gene expression, and (ii) growth on different carbohydrates results in distinct, substrate-specific gene expression signals capable of driving highly discriminatory differences between treatment groups. The application of rigorous bootstrapping to our hierarchical clustering results also revealed several cases of higher level clusters in which strong confidence was achieved. These dendrogram nodes (illustrated as white circles) indicate sets of growth conditions that yield gene expression patterns more like each other than like the patterns observed for other substrates. Two notable examples were xylan/arabinoxylan (which are structurally related and share the same xylan backbone) and L-fucose/L-rhamnose (which are known to be metabolized via parallel pathways in *E. coli* [44]).

Importantly, these findings suggested that by considering *in vitro* profiling data alongside *in vivo* expression data from the artificial community, it might be possible to identify the particular carbohydrates to which *B. cellulosilyticus* WH2 is exposed and responding within its gut environment. To explore this concept further, we compared expression of each gene in each condition to its expression on our control treatment, MM plus glucose (MM-Glc). The results revealed a dynamic PUL activation network in which some PULs were activated by a single substrate, some were activated by multiple substrates, and some were transcriptionally silent across all conditions tested. Of the 118 putative *susC*/*D* pairs in *B. cellulosilyticus* WH2 that we have used as markers of PULs, 30 were dramatically activated on one or more of the substrates tested; in these cases, both the *susC*- and *susD*-like genes in the cassette were up-regulated an average of >100-fold relative to MM-Glc across all technical replicates (Figure 5B). At least one *susC*/*D* activation signature was identified for every one of the 17 oligosaccharides and polysaccharides and for six of the 13 monosaccharides tested (Table S14). The lack of carbohydrate-specific PUL activation events for some monosaccharides (fructose, galactose, glucuronic acid, sucrose, and xylose) was expected, given that these loci are primarily dedicated to polysaccharide acquisition. Further inspection of gene expression outside of PULs disclosed that *B. cellulosilyticus* WH2 prioritizes use of its non-PUL-associated carbohydrate machinery, such as putative phosphotransferase system (PTS) components and monosaccharide permeases, when grown on these monosaccharides (Table S14).

Several carbohydrates activated the expression of multiple PULs. Growth on water-soluble xylan and wheat arabinoxylan produced significant up-regulation of five *susC*/*D*-like pairs (*BWH2_0865*/*6*, *0867*/*8*, *4044*/*5*, *4046*/*7*, and *4074*/*5*). No other substrate tested activated as many loci within the genome, again hinting at the importance of xylan and arabinoxylan to this strain's metabolic strategy *in vivo*. Cecal expression data from E_1_ showed that 15 of these activated PULs were expressed *in vivo* on one or both of the diets tested (see circles to the right of the heatmap in Figure 5B). In mice fed the polysaccharide-rich LF/HPP chow, *B. cellulosilyticus* WH2 up-regulates three *susC*/*D* pairs (*BWH2_2717*/*8*, *4044*/*5*, *4046*/*7*) whose expression is activated *in vitro* by arabinan and xylan/arabinoxylan. The three most significantly up-regulated *susC*/*D* pairs (*BWH2_1736*/*7*, *2514*/*5*, *4074*/*5*) in mice fed the HF/HS diet rich in sugar, corn starch, and maltodextrin are activated *in vitro* by amylopectin, ribose, and xylan/arabinoxylan, respectively. All three PULs identified as being up-regulated at the RNA level in LF/HPP-fed mice were also found to be up-regulated at the protein level (Figure 5B). Two of the three PULs up-regulated at the mRNA level in HF/HS-fed mice were up-regulated at the protein level as well. The presence of an amylopectin-activated PUL among these two loci is noteworthy, given the significant amount of starch present in the HF/HS diet. The up-regulation of four other PULs in HF/HS-fed animals was only evident in our LC-MS/MS data, reinforcing the notion that protein data both complement and supplement mRNA data when profiling microbes of interest.

Two of the five *susC*/*D* pairs activated by xylan/arabinoxylan form the four-gene cassette in the previously discussed PUL comprising *BWH2_4044*–*55* that is activated in mice fed the plant polysaccharide-rich chow (see Figure 4A). Another one of the five is the *susC*/*D* pair found in the PUL comprising *BWH2_4072*–*6* that is activated in mice fed the HF/HS “Western”-like chow (see Figure 4A). Thus, we have identified a pair of putative PULs in close proximity to one another on the *B. cellulosilyticus* WH2 genome that encode CAZymes with similar predicted functions, are subject to near-identical levels of specific activation by the same two polysaccharides (i.e., xylan, arabinoxylan) *in vitro*, but are discordantly regulated *in vivo* in a diet-specific manner. The highly expressed nature of these PULs in the diet environment where they are active, their shared emphasis on xylan/arabinoxylan utilization, and their tight regulation indicate that they are likely to be important for the *in vivo* success of this organism in the two nutrient environments tested. However, the reasons for their discordant regulation are unclear. One possibility is that in addition to being activated by xylan/arabinoxylan and related polysaccharides, these loci are also subject to repression by other substrates present in the lumen of the gut, and this repression is sufficient to block activation. Alternatively, the specific activators of each PUL may be molecular moieties shared by both xylan and arabinoxylan that do not co-occur in the lumenal environment when mice are fed the diets tested in this study.

### Prospectus

Elucidating generalizable “rules” for how microbiota operate under different environmental conditions is a substantial challenge. As our appreciation for the importance of the gut microbiota in human health and well-being grows, so too does our need to develop such rules using tractable experimental models of the gut ecosystem that allow us to move back and forth between *in vivo* and *ex vivo* analyses, using one to inform the other. Here, we have demonstrated the extent to which high-resolution DNA-, mRNA-, and protein-level analyses can be applied (and integrated) to study an artificial community of sequenced human gut microbes colonizing gnotobiotic mice. Our efforts have focused on characterizing community-level and species-level adaptation to dietary change over time and “leveraging” results obtained from *in vitro* assessments of individual species' responses to a panel of purified carbohydrates to deduce glycan exposures and consumption strategies *in vivo*. This experimental paradigm could be applied to any number of questions related to microbe–microbe, environment–microbe, and host–microbe interactions, including, for example, the metabolic fate of particular nutrients of interest (metabolic flux experiments), microbial succession, and biotransformations of xenobiotics.

Studying artificial human gut microbial communities in gnotobiotic mice also allows us to evaluate the technical limitations of current molecular approaches for characterizing native communities. For example, the structure of an artificial community can be evaluated over time at low cost using short read shotgun DNA sequencing data mapped to all microbial genomes within the community (COPRO-Seq). This allows for a much greater depth of sequencing coverage (i.e., more sensitivity) and much less ambiguity in the assignment of reads to particular taxa than traditional 16S rRNA gene-based sequencing. Short read cDNA sequences transcribed from total microbial community RNA can also often be assigned to the exact species and gene from which they were derived, and the same is also often true for peptides derived from particular bacterial proteins. However, substantial dynamic range in species/transcript/protein abundance within any microbiota, defined or otherwise, imposes limits on our ability to characterize the least abundant elements of these systems.

The effort to obtain a more complete understanding of the operations and behaviors of minor components of the microbiota is an area deserving of significant attention, given known examples of low-abundance taxa that play key roles within their larger communities and in host physiology [2],[45]. Developing such an understanding requires methods and assays that are collectively capable of assessing the structure and function of a microbiota at multiple levels of resolution. The need for high sensitivity and specificity in these approaches will become increasingly relevant as we transition towards experiments involving defined communities of even greater complexity, including bacterial culture collections prepared from the fecal microbiota of humans [46]. We anticipate that the study of sequenced culture collections transplanted to gnotobiotic mice will be instrumental in determining the degree to which physiologic or pathologic host phenotypes can be ascribed to the microbiota as well as specific constituent taxa.

The recent development of a low-error 16S ribosomal RNA amplicon sequencing method (LEA-Seq) and the application of this method to the fecal microbiota of 37 healthy adults followed for up to 5 years indicated that individuals in this cohort contained 195±48 bacterial strains representing 101±27 species [47]. Furthermore, stability follows a power-law function, suggesting that once acquired, most gut strains in a person are present for decades. New advances in the culturing of fastidious gut microbes may one day allow us to capture most (or all) of the taxonomic and functional diversity present within an individual's fecal microbiota as a clonally arrayed, sequenced culture collection, providing a perfectly representative and defined experimental model of their gut community. In the meantime, first-generation artificial communities of modest complexity such as the one described here offer a way of studying many questions related to the microbiota. However, the limited complexity and composition of our 12-species artificial community, and the way in which it was assembled in germ-free mice, make it an imperfect model of more complex human microbiota. Native microbial communities, for example, are subject to the influence of variables that are notably absent from this system, such as intraspecies genetic variability and exogenous microbial inputs. There are also taxa (e.g., Proteobacteria, Bifidobacteria) and microbial guilds (e.g., butyrate producers) typical of human gut communities that are absent from our defined assemblage that could be used to augment this system in order to improve our understanding of how their presence/absence influences a microbiota's response to diet and a spectrum of other variables of interest. These future attempts to systematically increase complexity should reveal what trends, patterns, and trajectories observed in artificial assemblages such as the one reported here map or do not map onto natural communities.

Finally, one of the greatest advantages of studying defined assemblages in mice is that they afford us the ability to interrogate the biology of key bacterial species in a focused manner. The artificial community we used in our experiments included *B. cellulosilyticus* WH2, a species that warrants further study as a model gut symbiont given its exceptional carbohydrate utilization capabilities, its apparent fitness advantage over many other previously characterized gut symbionts, and its genetic tractability. This genetic tractability should facilitate future experiments in which transposon mutant libraries are screened *in vivo* as one component of a larger artificial community in order to identify this strain's most important fitness determinants under a wide variety of dietary conditions. Identifying the genetic elements that allow *B. cellulosilyticus* to persist at the relatively high levels observed, regardless of diet, should provide microbiologists and synthetic biologists with new “standard biological parts” that will aid them in developing the next generation of prebiotics, probiotics, and synbiotics.

## Materials and Methods

### Ethics Statement

All experiments involving mice used protocols approved by the Washington University Animal Studies Committee in accordance with guidelines set forth by the American Veterinary Medical Association. Trained veterinarians from the Washington University Division of Comparative Medicine supervised all experiments. The laboratory animal program at Washington University is accredited by the Association for Assessment and Accreditation of Laboratory Animal Care International (AAALAC).

### *B. cellulosilyticus* WH2 Genome Sequencing

A strain of *B. cellulosilyticus* designated “WH2” (see Figure S1A,B) was isolated from a human fecal sample during an iteration of the Microbial Diversity Summer Course overseen by A. Salyers (University of Illinois, Urbana-Champaign) at the Marine Biological Laboratory (Woods Hole, MA). The genome of this isolate was sequenced using a combination of long-read and short-read technologies, yielding 51,819 plasmid and fosmid end reads (library insert sizes: 3.9, 4.9, 6.0, 8.0, and 40 kb; ABI 3730 platform), 333,883 unpaired 454 reads (FLX+ and XL+ chemistry), and 10 million unpaired Illumina reads (HiSeq; 42 nt read length). A hybrid assembly was constructed using MIRA v3.4.0 (method, *de novo*; type, genome; quality grade, accurate) with default settings [48],[49]. Gene calling was performed using the YACOP metatool [50]. Additionally, the four ribosomal RNA (rRNA) operons within the *B. cellulosilyticus* WH2 genome were sequenced individually to ensure high sequence accuracy in these difficult-to-assemble regions. Further details for the *B. cellulosilyticus* WH2 assembly are provided in Table S1.

### Bacterial Strains

Details regarding the 12 bacterial strains used in this study are provided in Table S4. Cells were grown in supplemented TYG (TYG_S_; [42]) at 37°C under anaerobic conditions in a Coy anaerobic chamber (atmosphere: 75% N_2_, 20% H_2_, 5% CO_2_). After reaching stationary phase, cells were pelleted by centrifugation and resuspended in TYG_S_ medium supplemented with 20% glycerol. Individual aliquots containing 400–800 µL of each cell suspension were stored at −80°C in 1.8 mL borosilicate glass vials with aluminum crimp tops. The identity of each species was verified prior to its use in experiments by extracting DNA from a frozen aliquot of cells, amplifying the 16S rRNA gene by PCR using primers 8F/27F (AGAGTTTGATCCTGGCTCAG; [51]) and 1391R (GACGGGCGGTGWGTRCA; [52]), sequencing the entire amplicon with an ABI 3730 capillary sequencer (Retrogen, Inc.), and comparing the assembled 16S rRNA gene sequence to the known reference sequence.

### Preparation of Strains for Oral Gavage

Details regarding the construction of each inoculum are provided in Table S3. The inocula used to gavage germ-free mice in each experiment were prepared either directly from frozen stocks (experiment 1, E_1_) or from a combination of frozen stocks and overnight cultures (experiment 2, E_2_). The recoverable cell density for each batch of frozen stocks used in inoculum preparation was determined prior to pooling, while the same values for overnight cultures were calculated after pooling. To do so, an aliquot of cells from each overnight culture or set of frozen stocks was used to prepare a 10-fold dilution series in phosphate-buffered saline (PBS), and each dilution series was plated on brain-heart-infusion (BHI; BD Difco) agar supplemented with 10% (v/v) defibrinated horse blood (Colorado Serum Co.). Plates were grown for up to 3 d at 37°C under anaerobic conditions in a Coy chamber, colonies were counted, and the number of colony-forming units per milliliter (CFUs/mL) was calculated. The volume of each cell suspension included in the final inoculum was normalized by its known or estimated viable cell concentration in an effort to ensure that no species received an early advantage during establishment of the artificial community in the germ-free animals. Total CFUs per gavage were estimated at 8.0×10^7^ and 4.2×10^8^ for experiments E_1_ and E_2_, respectively.

### Mice

Experiments were performed using protocols approved by the animal studies committee of the Washington University School of Medicine. For each experiment, two groups of 10–12-wk-old male germ-free C57BL/6J mice were maintained in flexible film gnotobiotic isolators under a strict 12 h light cycle, during which time they received sterilized food and water *ad libitum*. Animals were fasted for 4 h prior to gavage with 500 µL of a cell suspension inoculum containing the 12 sequenced, human gut-derived bacterial symbionts. After gavage, animals were maintained in separate cages throughout the course of the experiment. Fresh fecal pellets were periodically collected directly into screw-cap sample tubes that were immediately frozen in liquid nitrogen. At the time of sacrifice, the contents of each animal's cecum were divided into thirds and snap-frozen in liquid nitrogen for later use in DNA, RNA, and total protein isolations.

### Diets

Animals were subjected to dietary oscillations comprising three consecutive phases of 2 wk each (see Figure S3). Prior to inoculation, germ-free mice were maintained on a standard autoclaved chow diet low in fat and rich in plant polysaccharides (LF/HPP, B&K rat and mouse autoclavable chow #73780000, Zeigler Bros, Inc). Three days prior to inoculation, one group of germ-free animals was switched to a sterile “Western”-like chow high in fat and simple sugars (HF/HS, Harlan Teklad TD96132), while the other continued to receive LF/HPP chow. After gavage, each group of animals was maintained on its respective diet for 2 wk, after which each treatment group was switched to the alternative diet. Two weeks later, the mice were switched back to their original starting diet and were retained on this diet until the time of sacrifice.

### DNA and RNA Extraction

DNA and RNA were extracted from fecal pellets and cecal contents as previously described [11].

### Community Profiling by Sequencing (COPRO-Seq)

COPRO-Seq measurements of the proportional representation of all species present in each fecal/cecal sample analyzed were performed as previously described [11] using short-read (36 nt) data collected from an Illumina sequencer (data were generated using a combination of the Genome Analyzer I, Genome Analyzer II, and Genome Analyzer IIx platforms). After demultiplexing each barcoded pool, reads were trimmed to 25 bp and aligned to the reference genomes. An abundance threshold cutoff of 0.003% was set for determining an artificial community members' presence/absence, based on the proportion of reads from each experiment that were found to spuriously align to distractor reference genomes of bacterial species not included in this study. Normalized counts for each bacterial species in each sample were used to calculate a simple intrasample percentage. In order to make changes in abundance over time more easily comparable between species with significantly different relative abundances, these percentages were also in some cases normalized by the maximum abundance (%) observed for a given species across all time-points from a given animal. This transformation resulted in a value referred to as the percentage of maximum achieved (“PoMA”) that was used to evaluate which species were most/least responsive to dietary interventions.

### Ordination of COPRO-Seq Data Using QIIME

COPRO-Seq proportional abundance data were subjected to ordination using scripts found in QIIME v1.5.0-dev [53]. Data from both E_1_ and E_2_ were combined to generate a single tab-delimited table conforming to QIIME's early (pre-v1.4.0-dev) OTU table format. This pseudo-OTU table was subsequently converted into a BIOM-formatted table object that was used as the input for beta_diversity.py to calculate the pairwise distances between all samples using a Hellinger metric. PCoA calculations were performed using principal_coordinates.py. These coordinates and sample metadata were passed to make_3d_plots.py to generate PCoA plots. Plots shown are visualized using v2.21 of the KiNG software package [54].

### Metatranscriptomics

#### GeneChip

A custom Affymetrix GeneChip (“SynComm1”) with perfect match/mismatch (PM/MM) probe sets targeting 97.6% of the predicted protein-coding genes within the genomes of the 12 bacterial species in this study (plus three additional species not included in the model human gut microbiota) was designed and manufactured in collaboration with the Affymetrix chip design team. Control probes targeting intergenic regions from each genome were also tiled onto the array to allow detection of any contaminating gDNA. Hybridizations were carried out with 0.9–5.1 µg cDNA using the manufacturer's recommended protocols. Details regarding the design of this GeneChip are deposited under Gene Expression Omnibus (GEO) accession GPL9803.

Custom mask files were generated for each species on the GeneChip for the purpose of performing data normalization one species at a time. Normalization of raw intensity values was carried out in Affymetrix Microarray Suite (MAS) v5.0. MAS output was exported to Excel where advanced filtering was used to identify those probe sets called present in at least five of seven cecal RNA samples in at least one diet tested. Data from probe sets that did not meet these criteria (i.e., genes that were not expressed on either condition) were not included in subsequent analyses. Normalized, filtered data were evaluated using the Cyber-T web server [55] to identify differentially expressed genes. Genes were generally considered significantly differentially expressed in cases meeting the following three criteria: *p* < 0.01, PPDE(<*p*) ≥ 0.99, and |fold-change|≥ 2.

#### Microbial RNA-Seq

Methods for extracting total microbial RNA from mouse feces and cecal contents, depleting small RNAs (e.g., tRNA) and ribosomal RNA (5S, 16S, and 23S rRNA), and for converting depleted RNA to double-stranded cDNA were described previously [14]. Illumina libraries were prepared [11] from 26 fecal samples obtained from the second diet oscillation experiment (four animals, 6–7 time-points surveyed per animal), using 500 ng of input double-stranded cDNA/sample/library. RNA-Seq reads were aligned to the reference genomes using the SSAHA2 aligner [56]. Normalization of the resulting raw counts was performed using the DESeq package in R [57]. Raw counts derived from the metatranscriptome were normalized either at the community level (i.e., counts from all genes were included in the same table during normalization) for purposes of looking at community-level representation of functions (ECs) of interest, or at the species level (i.e., counts from each species were independently normalized) for purposes of looking at gene expression changes within individual species. Data adjustment (logarithmic transformation) and hierarchical clustering were performed using Cluster 3.0 [58] and GENE-E. Heatmap visualizations of expression data were prepared using JavaTreeview [59] and Microsoft Excel. The *B. cellulosilyticus* WH2 *in vitro* gene expression dendrogram presented was prepared using GENE-E. Bootstrap probabilities at each edge of the dendrogram were calculated using the “pvclust” package in R (10,000 replications). Clusters with bootstrap *p* values >0.95 were considered strongly supported and statistically significant.

### Metaproteomics

#### Sample Preparation

Cecal contents were collected from four mice and solubilized in 1 mL SDS lysis buffer (4% w/v SDS, 100 mM Tris·HCl (pH 8.0), 10 mM dithiothreitol (DTT)), lysed mechanically by sonication, incubated at 95°C for 5 min, and centrifuged at 21,000× *g*. Crude protein extracts were precipitated using 100% trichloroacetic acid (TCA), pelleted by centrifugation, and washed with ice-cold acetone to remove lipids and excess SDS. Protein precipitates were resolubilized in 500 µL of 8 M urea and 100 mM Tris·HCl (pH 8.0), reduced by incubation in DTT (final concentration of 10 mM) for 1 h at room temperature, and sonicated in an ice water bath (Branson (model SSE-1) sonicator; 20% amp; 2 min total (cycles of 5 s on, 10 s off)). An aliquot of each protein extract was quantified using a bicinchoninic acid (BCA)-based protein assay kit (Pierce). Protein samples (1 mg) were subsequently diluted with 100 mM Tris·HCl and 10 mM CaCl_2_ (pH 8.0) to a final urea concentration below 4 M. Proteolytic digestions were initiated with sequencing grade trypsin (1/100, w/w; Promega) and incubated overnight at room temperature. A second aliquot of trypsin was added (1/100) after the reactions were diluted with 100 mM Tris·HCl (pH 8.0) to a final urea concentration below 2 M. After incubation for 4 h at room temperature, samples were reduced by incubation in 10 mM DTT for 1 h at room temperature. Finally, the peptides were acidified (protonated) in 200 mM NaCl and 0.1% formic acid, filtered, and concentrated with a 10 kDa molecular weight cutoff spin column (Sartorius).

#### LC-MS/MS Data Collection

The peptide mixture from each mouse was analyzed in technical duplicate via two-dimensional liquid chromatography (LC)-MS/MS on a hybrid LTQ Orbitrap Velos mass spectrometer (Thermo Fisher Scientific). Peptides (∼100 µL per sample) were separated using a split phase 2D (strong-cation exchange (SCX) and C_18_ reverse phase (RP))-LC column over a 12-step gradient for each run. All MS analyses were performed in positive ion mode. Mass spectral data were acquired using Xcalibur (v2.0.7) in data-dependent acquisition mode for each chromatographic separation (22 h run). One precursor MS scan was acquired in the Orbitrap at 30K resolution followed by 10 data-dependent MS/MS scans (*m/z* 400–1,700) at 35% normalized collision energy with dynamic exclusion enabled at a repeat count of 1. MS/MS spectra were searched with SEQUEST (v.27; [60]) using the following settings: enzyme type = trypsin; precursor ion mass tolerance = 3.0 Da; fragment mass tolerance = 0.5 Da; fully tryptic peptides and those resulting from up to four missed cleavages only. All datasets were filtered with DTASelect (v1.9; [61]) using the following parameters: Xcorrs of 1.8, 2.5, and 3.5 for singly, doubly, and triply charged precursor ions; DeltCN ≥ 0.08; ≥2 fully tryptic peptides per protein.

A custom-built FASTA target-decoy database [62],[63] was generated and searched with SEQUEST at a peptide-level false positive rate (FPR) estimated at ≤0.5%. The database contained theoretical proteomes predicted from the genomes of the 12 bacterial species characterized in this study (see Tables S4 and S8), some diet components (e.g., rice and yeast), and common contaminants (e.g., keratins). Three additional theoretical bacterial proteomes predicted from the genomes of *Eubacterium rectale*, *Faecalibacterium prausnitzii*, and *Ruminococcus torques* were included as distractors (negative controls) that were not expected to be present in any of the samples analyzed. An *in silico* tryptically digested protein sequence database was also used to generate a theoretical peptidome of unique peptides within a mass range of 600–4,890 Da and ≤1 miscleavages.

#### Analysis of Proteomic Datasets

Spectral counts for each protein were normalized by either the total number of spectra collected for all species in a sample (normalization by community, “NBC”), or by the total number of spectra collected for all proteins from a given species (normalization by species, “NBS”). *p* values for each protein were calculated using the Mann–Whitney *U* test. To correct for multiple comparisons, *q* values were calculated using an optimized false discovery rate (FDR) approach with the “qvalue” package in BioConductor. Regardless of the normalization strategy employed, *p* and *q* values were only calculated for proteins with at least three valid runs, where a valid run was one with more than five spectral counts. In NBC data, *p* and *q* values were calculated for all proteins within the model metaproteome. In NBS data, *p* and *q* values were calculated for each species-specific set of proteins. Differences in spectral counts between treatment groups (diets) were calculated using group medians. A protein was designated as “UP”-regulated if both its *p* and *q* values were less than 0.05 and the spectral count difference between treatment groups was greater than 5. The same criteria were applied in the opposite direction for proteins labeled as “DOWN.” For proteins labeled “NULL,” there was insufficient evidence to report any significant difference between the two treatment groups. Finally, a protein was considered detected or “present” in a sample if at least four (raw) spectral counts were assigned to that protein when aggregating the results from the two runs (technical replications) performed on the sample.

### Phenotypic Screen for the Growth of *Bacteroides* spp. on Various Carbohydrates

The ability of *B. cellulosilyticus* WH2 and *B. caccae* ATCC 43185 to grow on a panel of 47 simple and complex carbohydrates was evaluated using a phenotypic array whose composition has been previously described [25]. Growth measurements were collected in duplicate (two wells per substrate) over the course of 3 d at 37°C under anaerobic conditions. A total of three independent experiments were performed for each species tested (*n* = 6 growth profiles/substrate/species). Total growth (A_tot_) was calculated from each growth curve as the difference between the maximum and minimum optical densities (OD_600_) observed (i.e., A_max_−A_min_). Growth rates were calculated as total growth divided by time (A_tot_/(t_max_−t_min_)), where t_max_ and t_min_ correspond to the time-points at which A_max_ and A_min_, respectively, were collected. Consolidated statistics from all six replicates for each of the 47 conditions tested for each species are provided in Table S11.

### Profiling *B. cellulosilyticus* WH2 Gene Expression During Growth in Defined MM Containing Various Carbohydrates

#### RNA-Seq

To characterize the impact of select mono- and polysaccharides on the *in vitro* gene expression of *B. cellulosilyticus* WH2, cells were cultured in MM supplemented with one of 31 distinct carbohydrates (for the formulation of MM and a list of the carbohydrates used as growth substrates, see Tables S12 and S13). After recovery from a frozen stock on BHI blood agar, a single colony was picked and inoculated into 5 mL of MM containing 5 mg/mL glucose (MM-Glc). Anaerobic conditions were generated within each individual culture tube using a previously described method [64] with the following modifications: (i) the cotton plug was lit and extinguished before being pushed below the lip of the culture tube, and (ii) 200 µL of saturated sodium bicarbonate was combined with 200 µL 35% (w/v) pyrogallate solution on top of the cotton plug before a bare rubber stopper was used to seal the tube. Cultures were grown overnight at 37°C. Twenty microliters of this “starter” culture were subsequently inoculated into a series of “acclimatization” cultures, each containing 5 mL of MM plus one of the 31 carbohydrates to be tested (5 mg/mL final concentration), and anaerobic culturing was carried out as above. This second round of culturing served two purposes: (i) it ensured cells were acclimated to growth on their new carbohydrate substrate prior to the inoculation of the final cultures that were harvested for RNA, and (ii) it provided an opportunity to obtain OD_600_ measurements indicating, for each carbohydrate, the range of optical densities corresponding to *B. cellulosilyticus* WH2's logarithmic phase of growth. Finally, 50 µL of each “acclimatization” culture were inoculated into triplicate 10 mL volumes of medium of the same composition, and the 90 “harvest” cultures were grown anaerobically at 37°C. At mid-log phase, 5 mL of cells were immediately preserved in Qiagen RNAprotect Bacteria Reagent according to the manufacturer's instructions. Cells were then pelleted, RNAprotect reagent was poured off, and the bacteria were stored at −80°C.

After thawing, while still cold, each bacterial cell pellet was combined with 500 µL Buffer B (200 mM NaCl, 20 mM EDTA), 210 µL of 20% SDS, and 500 µL of acid phenol∶chloroform∶isoamyl alcohol (125∶24∶1, pH 4.5). The pellet was resuspended by manual manipulation with a pipette tip and transferred to a 2 mL screwcap tube containing acid-washed glass beads (Sigma, 212–300 µm diameter). Tubes were placed on ice, bead-beaten for 2 min at room temperature (BioSpec Mini-Beadbeater-8; set to “homogenize”), placed on ice, and bead-beaten for an additional 2 min, after which time RNA was extracted as described above for fecal and cecal samples.

### Identification of Diet-Specific Fitness Determinants within the *B. cellulosilyticus* WH2 Genome Using Insertion Sequencing (INSeq)

Whole genome transposon mutagenesis of *B. cellulosilyticus* WH2 was performed using protocols originally developed for *B. thetaiotaomicron* [42],[46], with some modifications. Initial attempts to transform *B. cellulosilyticus* WH2 with the pSAM_Bt construct reported by Goodman et al. yielded very low numbers of antibiotic-resistant clones, which we attributed to poor recognition of one or more promoters in the mutagenesis plasmid. Replacement of the promoter driving expression of the transposon's erythromycin resistance gene (*ermG*) with the promoter for the gene encoding EF-Tu in *B. cellulosilyticus* WH2 (*BWH2_3183*) dramatically improved the number of resistant clones recovered after transformation. The resulting library consisted of 93,458 distinct isogenic mutants, with each mutant strain containing a single randomly inserted modified mariner transposon. Of all predicted ORFs, 91.5% had insertions covering the first 80% of each gene (mean, 13.9 distinct insertion mutants per ORF).

At 11 wk of age, male germ-free C57BL/6J mice (individually caged) were fed either a diet low in fat and rich in plant polysaccharides (LF/HPP) or high in fat and simple sugars (HF/HS). After a week on their experimental diet, animals received a single gavage containing the *B. cellulosilyticus* WH2 transposon library and 14 other species of bacteria (i.e., this artificial community consisted of the 12 species listed in Figure 1A, plus *B. thetaiotaomicron* 7330, *E. rectale* ATCC 33656, and *Clostridium symbiosum* ATCC 14940). After 16 d, fecal pellets were collected, and total fecal DNA was extracted.

500 ng of each fecal DNA extraction was diluted in 15 µL of TE buffer and digested with MmeI (4 U, New England Biolabs) in a 20 µL reaction supplemented with 10 pmoles of 12 bp DNA containing an MmeI restriction site (to improve the efficiency of restriction enzyme digestion) [42]. The reaction was incubated for 1 h at 37°C and then terminated (80°C for 20 min). MmeI-digested DNA was subsequently purified using 125 µL of AMPure beads (after washing the beads once with 100 µL of sizing solution (1.2 M NaCl and 8.4% PEG 8000)). The digested DNA was added to the beads and the solution incubated at room temperature for 5 min. Beads were pelleted with a magnetic particle collector (MPC), washed twice (each time using a mixture composed of 20 µL TE buffer (pH 7.0) and 100 µL sizing solution, with bead recovery via MPC after each wash), followed by two ethanol washes (180 µL 70% ethanol/wash) and air-drying for 10 min. Samples were resuspended in 18 µL TE buffer (pH 7.0), and DNA was removed after pelleting beads with the MPC. Ligation of adapters was performed in a 20 µL reaction that contained 16 µL of purified DNA, 1 µL of T4 Ligase (2000 U/µL; NEB), 2 µL 10× ligase buffer, and 10 pmol of barcoded adapter (incubation for 1 h at 16°C). Ligations were subsequently diluted with TE buffer (pH 7.0) to a final volume of 50 µL, mixed with 60 µL of AMPure beads, and incubated at room temperature for 5 min. Beads with bound DNA were pelleted using the MPC and washed twice with 70% ethanol as above. After allowing the ethanol to evaporate for 10 min, 35 µL of nuclease-free water was added, and the mixture was incubated at room temperature for 2 min before collecting the beads with the MPC. Enrichment PCR was performed in a final volume of 50 µL using 32 µL of the cleaned up sample DNA, 10 µL 10× *Pfx* amplification buffer (Invitrogen), 2 µL 10 mM dNTPs, 0.5 µL 50 mM MgSO_4_, 2 µL of 5 µM amplification primers (forward primer: 5′CAAGCAGAAGACGGCATACG3′, reverse primer: 5′AATGATACGGCGACCACCGAACACTCTTTCCCTACACGA3′), and 1.5 µL *Pfx* polymerase (2.5 U/µL; Invitrogen) (cycling conditions: denaturation at 94°C for 15 s; annealing at 65°C for 1 min; extension at 68°C for 30 s; total of 22 cycles). The 134 bp PCR product from each reaction was purified (4% MetaPhor gel; MinElute Gel Extraction Kit (Qiagen)) in a final volume of 20 µL and was quantified (Qubit, dsDNA HS Assay Kit; Invitrogen). Reaction products were then combined in equimolar amounts into a pool that was subsequently adjusted to 10 nM and sequenced (Illumina HiSeq 2000 instrument).

### Data Deposition

All short read Illumina data used for COPRO-Seq and RNA-Seq analyses, GeneChip data, and genome sequencing/assembly data are available through GEO SuperSeries GSE48537 and NCBI BioProject ID PRJNA183545. The draft genome assembly for *B. cellulosilyticus* WH2 has been deposited at DDBJ/EMBL/GenBank under accession number ATFI00000000. Raw MS data are available from the Dryad Digital Repository: http://dx.doi.org/10.5061/dryad.7fj1k.

## Supporting Information

Figure S1 **Phylogenetic relatedness of** ***B. cellulosilyticus*** **WH2 to other** ***Bacteroides*** **species.** (A) Near full-length 16S rRNA gene sequences from the *B. cellulosilyticus* WH2 isolate, its closest relatives (two strains of *Bacteroides xylanisolvens*, three strains of *Bacteroides intestinalis*, and the type strain of *B. cellulosilyticus*), and *Parabacteroides distasonis* (the latter was included as an outgroup) were aligned against the SILVA SEED using the SINA aligner [65]. The 5′ and 3′ ends of the resulting multiple sequence alignment were trimmed to remove ragged edges, and the final alignment was used to construct an approximately maximum-likelihood phylogenetic tree using FastTree v2.1.4 [66]. Sequences in the trimmed alignment used to generate the tree shown correspond to bases 22–1498 of the *Escherichia coli* 16S rRNA gene [67]. Parenthetical identifiers indicate the locus tag (for *B. cellulosilyticus* WH2, whose genome contains four copies of the 16S rRNA gene) or GenBank accession number (for all other strains) of each sequence included in the phylogenetic analysis. (B) Identity matrix summarizing the pairwise similarities (as % nucleotide sequence identity) for all 16S rRNA gene sequences used to construct the tree shown in panel (A).
(EPS)

Figure S2 **Representation of all putative GH families identified in the** ***B. cellulosilyticus*** **WH2 genome compared to their representation in other sequenced Bacteroidetes species.** Enumeration of the GH repertoire of *B. cellulosilyticus* WH2 relative to (A) the six other Bacteroidetes species included in the artificial microbial community described in Figure 1A, and (B) the 86 Bacteroidetes currently annotated in the CAZy database. GH numbers in red signify CAZy families whose representation is greater in *B. cellulosilyticus* WH2 than in any of the other Bacteroidetes to which it is being compared. An asterisk following a GH family number indicates that genes encoding proteins from that family were found exclusively in the *B. cellulosilyticus* WH2 genome. In (B), GH family numbers are ordered from left to right and from top to bottom by their average representation within the 87 Bacteroidetes genomes included in the analysis.
(TIF)

Figure S3 **Design and sampling schedule for experiments E_1_ and E_2_.** In each experiment, two groups of C57BL/6J germ-free mice were gavaged at 10–12 wk of age with a 12-member artificial human gut microbial community (the day of gavage, referred to as day 0, is denoted by a large black arrow). Over time, animals were fed diets low in fat and high in plant polysaccharides (LF/HPP, bold green) or high in fat and simple sugar (HF/HS, bold orange) in alternating fashion. Fecal pellets and cecal contents were collected as indicated for profiling community membership and gene expression (sample types are denoted by a circle's color and the methods applied to each sample are indicated in parentheses within the sample key). Values shown along the time course indicate the number of days since gavage of the artificial community into germ-free animals.
(EPS)

Figure S4 **COPRO-Seq analysis of the proportional representation of component taxa in the 12-member artificial community as a function of time after colonization of gnotobiotic mice and the diet they were consuming.** (A) Average DNA yields from fecal and cecal samples collected from each treatment group in experiment E_1_. (B) DNA yields from samples collected in experiment E_2_. (C) COPRO-Seq quantitation of the 12 bacterial species comprising the assemblage used to colonize germ-free mice in experiments E_1_ and E_2_. Vertical dashed lines at days 14 and 28 denote time-points at which diets were switched. Panels (A–C) share a common key, provided in the upper right. Circles and triangles denote samples from experiments E_1_ and E_2_, respectively. Cecal sample data points (obtained at sacrifice on day 42 of the experiment) are plotted as for fecal sample data, but with inverted colors (i.e., colored outline, solid black fill). For all panels (A–C), data shown are mean ± SEM.
(EPS)

Figure S5 **Further COPRO-Seq analysis of the relative abundance of components of the 12-member bacterial community as a function of diet and time.** (A) Plot of the ordination results for experiment 1 (E_1_) from the PCoA described in Figure 1B. COPRO-Seq data from E_1_ and E_2_ were ordinated in the same multidimensional space. For clarity, only data from E_1_ are shown (for the E_2_ PCoA plot, see Figure 1B). Color code: red/blue, feces; pink/cyan, cecal contents. (B) Heatmap representation of the relative abundance data from E_1_ normalized to each species' maximum across all time-points within a given animal (“Percentage of maximum achieved (PoMA)”). Each heatmap cell denotes the mean for one treatment group (*n* = 7 animals), and each treatment group is shown as its own heatmap.
(EPS)

Figure S6 **GeneChip profiling of the cecal metatranscriptome in mice fed different diets.** (A) Venn diagram illustrating the number of bacterial genes whose expression was scored as “present” (i.e., detectable in ≥5 of 7 animals), only in mice that were consuming the plant polysaccharide-rich LF/HPP diet, only in mice that were consuming a “Western”-like HF/HS diet, or in both groups. (B) Overview of the diet-specificity of CAZyme gene expression in the 12-member model microbiota and in four prominent taxa that maintained a proportional representation in the cecal microbiota that was >5% on each diet.
(EPS)

Figure S7 **Dissecting the** ***in vivo*** **expression of EC 3.2.1.8 (endo-1,4-β-xylanase).** (A) Gene expression in E_2_ fecal samples was evaluated by microbial RNA-Seq. After data from all 12 species in the model human gut microbiome were binned by EC number annotation and normalized (i.e., data were “community-normalized” at the level of ECs), a significant decrease in the representation of EC 3.2.1.8 in the metatranscriptome was observed when comparing the final time-point of the first diet phase (day 13, LF/HPP diet) and the final time-point of the second diet phase (day 27, HF/HS diet) (Mann–Whitney *U* test, *p* = 0.03). (B) Transcribed *B. cellulosilyticus* WH2 genes account for >99% of community-normalized RNA-Seq counts assignable to EC 3.2.1.8 (note how counts at the community level in panel (A) compare to those attributable to *B. cellulosilyticus* WH2 in panel (B)). Thus, *B. cellulosilyticus* WH2 essentially dictates the degree to which expressed endo-1,4-β-xylanase genes are represented within the metatranscriptome. (C) *B. cellulosilyticus* WH2 contributes a greater number of community-normalized RNA-Seq counts to the metatranscriptome in LF/HPP-fed mice than in HF/HS-fed animals. (D) When *B. cellulosilyticus* WH2 gene expression data are normalized independently of data from other taxa (i.e., when data are “species-normalized”), statistically significant increases in the representation of EC 3.2.1.8 within the *B. cellulosilyticus* WH2 transcriptome become apparent in HF/HS-fed mice. (E) Breakdown of the total species-normalized counts in panel (D) by the *B. cellulosilyticus* WH2 gene from which they were derived. For all panels (A–E), mean values ± SEM are shown. Means for all panels were calculated from data from four animals at each time-point, except day 26 (*n* = 2). In each of the first four panels (A–D), the differences between day 13 and day 27 were deemed statistically significant by Mann–Whitney *U* test (*p* = 0.03 for each of the four tests performed).
(EPS)

Figure S8 **Shotgun metaproteomic analysis of cecal samples from gnotobiotic mice colonized with the 12-member artificial community.** (A) Each species' theoretical proteome was subjected to *in silico* trypsinization (see *Materials and Methods*). Of the resulting peptides, those specific to a single protein within our database of all predicted proteins encoded by the genomes of the 12 assemblage members, the mouse, and three bacterial “distractors” (*E. rectale*, *F. prausnitzii*, and *R. torques*) were classified as “unique,” while all others were considered “nonunique.” The “unique” fraction of a species' predicted peptides indicates how many can be unambiguously traced back to a single protein of origin if detected by LC-MS/MS. (B) Comparison of the average relative cecal abundance of each assemblage member (dark gray bars) with the percentage of proteins within its theoretical proteome that were detected by LC-MS/MS (red bars), and the percentage of all genes within its genome whose expression was detected using our custom GeneChip (light gray bars). Data shown are mean values ± SEM. (C) Scatter plots illustrating the Pearson correlation coefficient (*r*) between log-transformed averages of diet-specific fold-differences in expression as determined by GeneChip assay (RNA, *x*-axis) and LC-MS/MS (protein, *y*-axis) in E_1_. Data points within the black scatter plot represent the 448 *B. cellulosilyticus* WH2 genes for which reliable quantitative data could be obtained for animals in both diet treatment groups for both the GeneChip and LC-MS/MS assays (i.e., any gene for which a signal could not be detected on at least one diet treatment in at least one assay was excluded). Scatter plots in color represent the results of correlation analyses performed on subsets of genes within the black plot whose KEGG annotations fall within particular functional categories, including “Translation” (*r* = 0.03, 59 genes), “Energy metabolism” (*r* = 0.36, 58 genes), “Amino acid metabolism” (*r* = 0.48, 67 genes), and “Carbohydrate metabolism” (*r* = 0.69, 110 genes). For both (B) and (C), *n* = 2 mice per treatment group (4 mice total).
(EPS)

Table S1 **Sequencing statistics for** ***B. cellulosilyticus*** **WH2.**
(XLSB)

Table S2 ***B. cellulosilyticus*** **WH2 genome features with relevance to carbohydrate metabolism.**
(XLSB)

Table S3 **Composition of the 12-member artificial community inoculated by oral gavage into germ-free animals.**
(XLSB)

Table S4 **Bacterial strains included in this study.**
(XLSB)

Table S5 **COPRO-Seq quantitation of the relative abundances of artificial community members over time.**
(XLSB)

Table S6 **GeneChip measurements of cecal gene expression for the 12 bacterial species comprising the artificial human gut microbial community studied in experiment E_1_.**
(XLSB)

Table S7 **List of EC numbers whose representation within the fecal metatranscriptome is significantly impacted by diet.**
(XLSB)

Table S8 **Summary of theoretical peptidome statistics.**
(XLSB)

Table S9 **Number of proteins detected within each cecal sample for each species in our custom SEQUEST database.**
(XLSB)

Table S10 **Raw and normalized MS/MS spectral counts for detectable proteins in E_1_ cecal samples.**
(XLSB)

Table S11 **Growth of** ***B. cellulosilyticus*** **WH2 and** ***B. caccae*** **on a panel of structurally diverse carbohydrates.**
(XLSB)

Table S12 **Preparation of minimal medium for** ***in vitro*** **gene expression profiling of** ***B. cellulosilyticus*** **WH2.**
(XLSB)

Table S13 **Carbohydrate substrates tested during** ***in vitro*** **gene expression profiling of** ***B. cellulosilyticus*** **WH2.**
(XLSB)

Table S14 **RNA-Seq gene expression values for** ***B. cellulosilyticus*** **WH2 grown** ***in vitro*** **on 31 simple and complex saccharides.**
(XLSB)

Text S1 **Supplementary results.**
(DOCX)

## Abbreviations

CE: carbohydrate esterase
COPRO-Seq: community profiling by sequencing
EC: enzyme commission
ECF: extracytoplasmic function
GH: glycoside hydrolase
GT: glycosyltransferase
HF/HS: high-fat/high-sugar
HTCS: hybrid two-component system
INSeq: insertion sequencing
LF/HPP: low-fat/high-plant polysaccharide
MM: minimal medium
MPC: magnetic particle collector
NBC: normalization by community
NBS: normalization by species
PCoA: principal coordinates analysis
PL: polysaccharide lyase
PoMA: percentage of maximum achieved
PSMs: peptide-spectrum matches
PTS: phosphotransferase system
PUL: polysaccharide utilization locus
RFO: raffinose family oligosaccharide
