# Supplementary figures and images for: Effects of Diet on Resource Utilization by a Model Human Gut Microbiota Containing Bacteroides cellulosilyticus WH2, a Symbiont with an Extensive Glycobiome
Source: PLoS Biol. 2013 Aug 20;11(8):e1001637. doi: 10.1371/journal.pbio.1001637 (PMC3747994; doi:10.1371/journal.pbio.1001637)

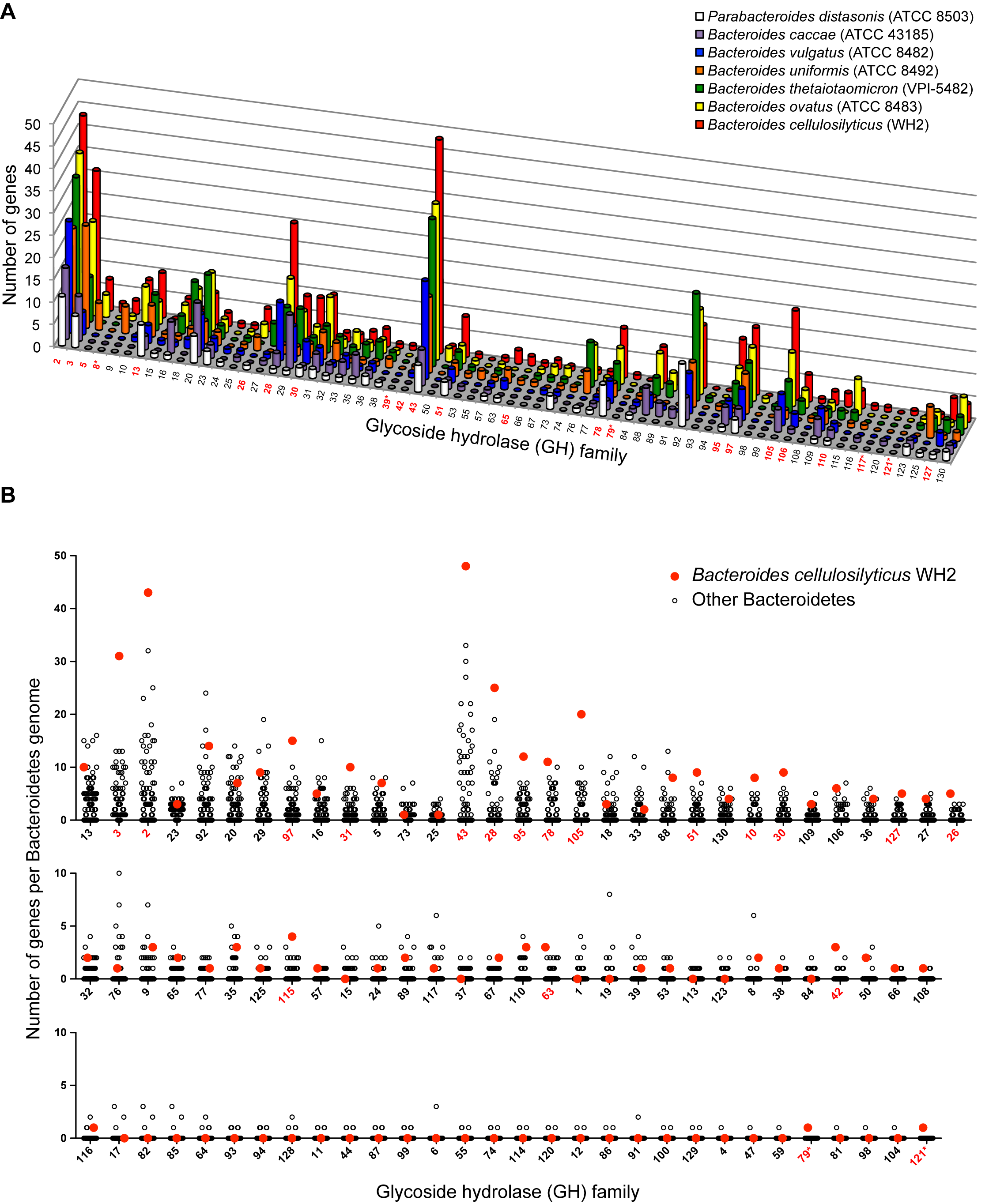

Supplement: Figure S2 — Representation of all putative GH families identified in the B. cellulosilyticus WH2 genome compared to their representation in other sequenced Bacteroidetes species. Enumeration of the GH repertoire of B. cellulosilyticus WH2 relative to (A) the six other Bacteroidetes species included in the artificial microbial community described in Figure 1A, and (B) the 86 Bacteroidetes currently annotated in the CAZy database. GH numbers in red signify CAZy families whose representation is greater in B. cellulosilyticus WH2 than in any of the other Bacteroidetes to which it is being compared. An asterisk following a GH family number indicates that genes encoding proteins from that family were found exclusively in the B. cellulosilyticus WH2 genome. In (B), GH family numbers are ordered from left to right and from top to bottom by their average representation within the 87 Bacteroidetes genomes included in the analysis. (TIF) [file pbio.1001637.s002.tif]
