# Supplementary material for: Effects of Diet on Resource Utilization by a Model Human Gut Microbiota Containing Bacteroides cellulosilyticus WH2, a Symbiont with an Extensive Glycobiome
Source: PLoS Biol. 2013 Aug 20;11(8):e1001637. doi: 10.1371/journal.pbio.1001637 (PMC3747994; doi:10.1371/journal.pbio.1001637)
Supplement: Text S1 — Supplementary results. (DOCX) [file pbio.1001637.s023.docx]

**Supplementary Results**

**Evaluating the carbohydrate utilization capabilities and preferences of *B. caccae*, a HF/HS diet-adapted species.**

Comparing CAZyme expression between three diet-insensitive *Bacteroides* spp. (*B. thetaiotaomicron*, *B. vulgatus*, and *B. cellulosilyticus* WH2) and HF/HS-favoring *B. caccae* revealed that these two groups have dissimilar profiles. While diet-insensitive strains express many CAZymes on both diets, and roughly equal percentages of their encoded CAZymes in a diet-specific manner, *B. caccae*'s CAZyme utilization is heavily skewed (**Fig. S6B**). While 19% of *B. caccae* CAZymes were expressed in mice regardless of the diet consumed, an additional 28% of this species' predicted CAZymes were expressed in animals consuming the HF/HS diet. In contrast, *B. caccae* expressed only 1% of its predicted CAZymes in a LF/HPP diet-specific manner.

Phenotypic characterization of *B. caccae* on the same carbohydrate growth array we used to characterize *B. cellulosilyticus* WH2's substrate utilization capabilities revealed significant deficiencies in *B. caccae*'s ability to utilize many simple and complex sugars (**Table S11**). Of particular note was its complete lack of starch utilization (as evidenced by its inability to grow on amylopectin derived from both potato and maize, as well as dextran and pullulan) and its inability to utilize any type of hemicellulose or β-glucan tested. These deficiencies strongly contrast the strong growth we observed for *B. cellulosilyticus* WH2 when it was grown on several such compounds. A complete comparison of the growth capabilities of *B. cellulosilyticus* WH2 and *B. caccae* reveals the striking fact that with the exception of one monosaccharide (N-acetylneuraminic acid), *B. cellulosilyticus* WH2 growth outperforms that of *B. caccae* on every carbohydrate tested.
